# Dimethyl fumarate impairs differentiated B cells and fosters central nervous system integrity in treatment of multiple sclerosis

**DOI:** 10.1111/bpa.12711

**Published:** 2019-03-05

**Authors:** Jan Traub, Sarah Traffehn, Jasmin Ochs, Silke Häusser‐Kinzel, Schirin Stephan, Robert Scannevin, Wolfgang Brück, Imke Metz, Martin S. Weber

**Affiliations:** ^1^ Institute of Neuropathology University Medical Center Göttingen Germany; ^2^ Department of Neurology University Medical Center Göttingen Germany; ^3^ Neurology Discovery Group Biogen Idec Cambridge MA

**Keywords:** B cell, CNS demyelination, dimethyl fumarate, multiple sclerosis, repair

## Abstract

In multiple sclerosis (MS), the effect of dimethyl fumarate (DMF) treatment is primarily attributed to its capacity to dampen pathogenic T cells. Here, we tested whether DMF also modulates B cells, which are newly recognized key players in MS, and to which extent DMF restricts ongoing loss of oligodendrocytes and axons in the central nervous system (CNS). Therefore, blood samples and brain tissue from DMF‐treated MS patients were analyzed by flow cytometry or histopathological examination, respectively. Complementary mechanistic studies were conducted in inflammatory as well as non‐inflammatory CNS demyelinating mouse models. In this study, DMF reduced the frequency of antigen‐experienced and memory B cells and rendered remaining B cells less prone to activation and production of pro‐inflammatory cytokines. Dissecting the functional consequences of these alterations, we found that DMF ameliorated a B cell‐accentuated experimental autoimmune encephalomyelitis model by diminishing the capacity of B cells to act as antigen‐presenting cells for T cells. In a non‐inflammatory model of toxic demyelination, DMF limited oligodendrocyte apoptosis, promoted maturation of oligodendrocyte precursors and reduced axonal damage. In a CNS biopsy of a DMF‐treated MS patient, we equivalently observed higher numbers of mature oligodendrocytes as well as a reduced extent of axonal damage when compared to a cohort of treatment‐naïve patients. In conclusion, we showed that besides suppressing T cells, DMF dampens pathogenic B cell functions, which probably contributes to its clinical effectiveness in relapsing MS. DMF treatment may furthermore limit chronically ongoing CNS tissue damage, which may reduce long‐term disability in MS apart from its relapse‐reducing capacity.

**Abbreviations**
APC: antigen‐presenting cell
APP: amyloid precursor protein
CFSE: carboxyfluorescein succinimidyl ester
CNS: central nervous system
CpG: CpG oligodeoxynucleotides
DMF: dimethyl fumarate
EAE: experimental autoimmune encephalomyelitis
EDSS: expanded disability status scale
ELISA: enzyme‐linked Immunosorbent Assay
GM‐CSF: granulocyte‐macrophage colony‐stimulating factor
H&E: hematoxylin and eosin
HPMC: hydroxypropyl methylcellulose
IFN: interferon
IL: interleukin
LFB/PAS: Luxol Fast Blue and periodic acid Schiff
MACS: magnetic‐activated cell sorting
MHC: major histocompatibility complex
MMF: monomethyl fumarate
MOG: myelin oligodendrocyte glycoprotein
MS: multiple sclerosis
Nrf2: nuclear factor erythroid‐derived 2‐related factor 2
OPC: oligodendrocyte precursor cells
PBMC: peripheral blood mononuclear cells
PMA: phorbol 12‐myristate 13‐acetate
Th: T helper
TNF: tumor necrosis factor

## Introduction

Dimethyl fumarate (DMF) is an oral formulation approved for the treatment of relapsing‐remitting multiple sclerosis (MS) 10, 13, 44. Its clinical effectiveness is primarily attributed to a modulating effect on T cells 11, 20. DMF has been shown to decrease the total number of circulating T cells, with a disproportionate reduction of the CD8^+^ subset 47. While the proportion of naïve and regulatory cells is enriched, memory T cell frequency and the total numbers of differentiated T helper (Th)1 and Th17 cells are diminished 35, 50. Besides these presumably direct effects on T cells 11, 48, DMF dampens the pro‐inflammatory activity of T cell‐activating antigen‐presenting cells (APC) such as monocytes and dendritic cells 39, 42, 45.

It was recently proposed that the key molecular mechanism of DMF may be a general downregulation of aerobic glycolysis, especially in cells with a high metabolic turnover providing a plausible explanation why DMF primarily affects activated immune cells such as effector and memory T cells 26. Apart from this, DMF is assumed to diminish inflammatory responses by various other properties, including activation of the nuclear factor erythroid‐derived 2‐related factor 2 (Nrf2) 32 and agonistic signaling at the hydroxycarboxylic acid receptor 2 5.

Based on these unselective mechanisms of action, we hypothesized that other immune cells, such as B lymphocytes, should be affected by DMF as well. B cells are increasingly recognized as key player in MS pathogenesis, a concept that was boosted by the enormous success of B cell‐depleting therapies in MS 17, 18, 24. Most plausibly, this benefit is achieved by abrogating two key functions of B cells, potent APC function 49 as well as provision of pro‐inflammatory cytokines 2. While it has been reported that DMF reduces the total number of B cells in the blood of MS patients 47 with a predominant decline in differentiated cells 19, 31, 34, 36, 46, the functional consequences remained to be elucidated. Accordingly, we set out to investigate which B cell subsets are most vulnerable to DMF treatment, how they are affected regarding activation and differentiation and whether these changes may translate into a modified APC capacity and cytokine profile.

Again, based on its broad mechanism of action, we further hypothesized that the small molecule DMF, or rather its active metabolite monomethyl fumarate (MMF) may also influence central nervous system (CNS) resident cells, such as microglia, astrocytes and oligodendrocytes, possibly reducing CNS tissue destruction. The pathological interplay of these cells is assumed to cause a steady chronic progression and accumulation of persisting disability in MS 30. This CNS‐intrinsic process likely initiates clinically unrecognized in earlier disease stages that are dominated by acute relapses and yet determines the longer‐term outcome of MS 29. It is thus of outmost importance to understand if DMF is also capable of dampening this progression‐driving cellular circuit within the CNS. Therefore, we studied to what extent DMF may limit CNS demyelination and axonal loss independent of its effects on the peripheral immune system.

## Methods and Materials

### Study subjects

Relapsing‐remitting MS patients were enrolled after written informed consent; protocols were approved by the resident ethical review committee (#19/09/10 and #3/4/14). Blood was collected from DMF‐treated and non‐DMF‐treated patients. The brain biopsy was obtained from one DMF‐treated MS patient for diagnostic reasons. The patient had developed new extensive gadolinium enhancing lesions, while the cerebrospinal fluid analysis remained inconclusive (Table 1).

**Table 1:** Characteristics of the patient cohorts. Multiple sclerosis (MS) patients of the control cohort had never been treated with dimethyl fumarate (DMF) when phlebotomy was performed, while DMF‐treated MS patients had taken DMF for at least three months before sampling. For longitudinal analysis, two samples of an individual patient were collected, one before and one after DMF treatment initiation. The biopsied control MS cohort had not received any immune modulatory or immune suppressive therapy. Abbreviations: y = years; SD = standard deviation; m = months; EDSS = expanded disability status scale.

	Horizontal		Longitudinal	MS biopsies	
	Control	DMF	(Switch to DMF)	Control	DMF
Number of patients	24	25	6	18	1
Age (y) (mean ± SD)	37.2 ± 9.8	35.4 ±11.1	34.3 ± 9.4	30.1 ± 3.8	52
Female sex (%)	75.0	48.0	50.0	58.8	0
EDSS score (mean ± SD)	2.2 ± 2.0	1.4 ± 1.2	1.8 ± 1.3	3.5 ± 2.0	2.0
MS since (y) (mean ± SD)	7.7 ± 10.0	3.0 ± 3.2	10.8 ± 7.4	0.2 ± 0.4	0.3
DMF since (m) (mean ± SD)	–	12.8 ± 7.5	7.6 ± 2.2	–	2.5
Previous treatment (cases):					
Natalizumab	2	1	0	0	0
Interferon beta	5	5	1	0	0
Glatiramer acetate	2	6	2	0	0

### Cell preparation

Human peripheral blood mononuclear cells (PBMC) were isolated by Ficoll density gradient centrifugation (Biochrom, Berlin, Germany). For analysis of activation markers and cytokine production, cells were incubated with 2 µg/mL CpG oligodeoxynucleotides (CpG) or 100 pg/mL lipopolysaccharide (both Sigma Aldrich, St. Louis, MO, USA) for 20 h at 37°C.

### Flow cytometry

After Zombie™ dye and Fc‐block (both BioLegend, San Diego, CA, USA) pre‐incubation, PBMC were stained with anti‐human CD14‐FITC, CD19‐PerCP‐Cy5.5, CD40‐PE‐Dazzle, CD69‐FITC, CD86‐BV421, CD95‐PE (all BD Bioscience, NJ, USA), CD4‐PE‐Cy7, CD8‐PE, CD14‐PE‐CF594, CD19‐FITC, CD20‐APC‐Cy7, CD24‐PerCp‐Cy5.5, CD25‐BV605, CD27‐PacificBlue, CD38‐FITC, CD80‐PE‐Cy7 and major histocompatibility complex (MHC)‐II‐APC (all BioLegend, CA, USA). Cytokines were evaluated after adding 1‐µL Golgi‐Plug (BioLegend) to CpG‐stimulated cells for 4 h; 500 ng/mL ionomycin and 20 ng/mL phorbol 12‐myristate 13‐acetate (PMA; Sigma Aldrich, MO) were added after 2 h. Cells were stained with anti‐human interleukin‐ (IL‐)6‐FITC, tumor necrosis factor (TNF)‐A700 and IL‐10‐PE‐CF594 (all BioLegend).

### Histopathology of human biopsies

(Immuno‐)histochemical studies were performed on paraffin‐embedded brain tissue according to published criteria 3. Early active demyelinating lesion areas were analyzed for acute axonal damage (anti‐amyloid precursor protein (APP), 1:2000, Merck Millipore, Burlington, MA, USA) and oligodendrocyte numbers (anti‐Olig2, 1:300, IBL, Hamburg, Germany; anti‐Nogo‐A, 1:50, Santa Cruz, Dallas, TX, USA).

### Mice

C57BL/6J mice were obtained from Charles River (Sulzfeld, Germany). Myelin oligodendrocyte glycoprotein (MOG) peptide_35‐55 _T cell receptor transgenic 2D2 mice were bred in the resident Central Animal Facility. All animal experiments were approved by the Lower Saxony authorities for animal experimentation (AZ G 13/1334).

### Experimental autoimmune encephalomyelitis (EAE)

Mice were subcutaneously injected with 100 µg recombinant mouse MOG protein_1‐117_ (provided by C.C.A. Bernard 6) emulsified in complete Freund’s adjuvant. Directly and 48 h after immunization, mice were intraperitoneally injected with 200 ng pertussis toxin (List biological laboratories, Campbell, CA, USA) 49.

### Cuprizone treatment of mice

Demyelination was induced by feeding mice with 0.25% cuprizone (Sigma Aldrich, MO) mixed into standard chow ad libitum for 7 days or 6 weeks. In an interventional setting, mice were fed with cuprizone for 5 weeks, followed by 3 days with normal chow.

### DMF treatment of mice

About 15 mg/kg DMF was administered via oral gavage as 10 µL/g body weight dissolved in a 0.8% hydroxypropyl methylcellulose (HPMC) solution (both Biogen Idec, Cambridge, MA, USA) twice daily. Controls received 0.8% HPMC solution.

### Flow cytometry of murine cells

Murine spleens and lymph nodes were dissected and passed through 70 µm strainers. After pre‐incubation with Zombie™ dye and Fc‐block (BioLegend), cells were stained with antibodies against B220‐FITC, B220‐PE‐Cy7, CD19‐FITC, CD25‐APC, CD69‐PerCP‐Cy5.5, CD95‐PE (all BD Bioscience), CD4‐PE, CD8‐FITC, CD11b‐BV510, CD21‐FITC, CD23‐APC, CD40‐PE, CD80‐PerCP‐Cy5.5, CD86‐PE and MHC‐II‐PacificBlue (all BioLegend). T cells were stained for interferon (IFN)‐γ‐APC (BioLegend) and IL‐17A‐PE (BD Bioscience) after 4‐h incubation with 50 ng/mL PMA, 0.5 μg/mL ionomycin and 1 μL/mL Golgi‐Plug; regulatory T cells were determined by intracellular staining for Foxp3‐PE (BD Bioscience).

### T cell proliferation assays

0.2x10^5^ magnetic‐activated cell sorting (MACS)‐purified (Pan TC Isolation Kit, Miltenyi, Germany) carboxyfluorescein succinimidyl ester (CFSE)‐stained (BioLegend) T cells from naïve C57BL/6 mice treated with DMF or control were plated in 96‐well plates and stimulated with 0, 0.125 or 0.25 µg/mL LEAF™ purified anti‐mouse CD3 (BioLegend) and 0.5 µg/mL LEAF™ purified anti‐mouse CD28 antibodies (BioLegend) for 72 h. 0.5 × 10^6^ MACS‐purified B cells (Biotin mouse lineage panel, BD Biosciences, Franklin Lakes, NJ, USA) from spleens of DMF‐ or control‐treated immunized C57BL/6 mice were plated in 96‐well plates and co‐cultured with 0.2 × 10^5^ MACS‐purified CFSE‐stained 2D2 T cells, stimulated with MOG protein_1‐117_ and evaluated 65 h thereafter.

### MOG protein_1‐117_ binding assay

0.5 × 10^6^ MACS‐purified B cells were incubated for 2 h with 20 µg/mL MOG protein_1‐117_ previously labeled using the DyLight‐405 labeling kit (Thermo Fisher Scientific, Waltham, MA, USA).

### Detection of cytokines using enzyme‐linked immunosorbent assay (ELISA)

IFN‐γ and granulocyte‐macrophage colony‐stimulating factor (GM‐CSF) were detected using paired monoclonal antibodies (ELISA MAX™ standard set, DuoSet, R&D Systems, Minneapolis, MN, USA). For data analysis, iMark™ microplate reader was used (Bio‐Rad Laboratories Inc., Hercules, CA, USA).

### Measuring monomethyl fumarate in plasma and brain

The laboratory of Rob Scannevin measured MMF (Biogen, MA, USA). About 25 μL of either plasma or tissue homogenate samples were extracted by protein precipitation with acetonitrile containing 4C13‐MMF as the internal standard. Homogenization solution (plasma with 12.5 mg/mL of sodium fluoride) was added to the tissue and samples were homogenized at 6.5 m/s for 60 s on a Fast Prep Tissue Homogenizer prior to protein precipitation. The concentrations of MMF was determined using qualified LC‐MS/MS assays in the respective matrices. Data collections and integrations were accomplished using an API 5500 triple quadrupole mass spectrometer with a turbo ion spray interface (AB Sciex, Redwood City, CA, USA). The peak area ratios of MMF relative to its internal standard were used to construct a standard curve using a quadratic regression with a 1/×2 weighting.

### Histology of murine samples

Mice were perfused with phosphate‐buffered saline and 4% paraformaldehyde. Brain and spinal cord were paraffin‐embedded and transverse sections were stained with Luxol Fast Blue and periodic acid Schiff (LFB/PAS) or hematoxylin and eosin (H&E). CNS tissue was further evaluated after labeling with antibodies against APP (1:2000 dilution, Merck Millipore), B220 (1:200, BD Bioscience), caspase‐3 (1:150, BD Bioscience), CD3 (1:200, Bio‐Rad Laboratories Inc., CA, USA), Mac‐3 (1:200, BD Bioscience), Nogo‐A (1:50, Santa Cruz) and Olig2 (1:300, IBL).

### Statistical analysis

Data were analyzed using BD FacsDiva, FlowJo 10.1 and GraphPad Prism 6 and tested for normal distribution with the D’Agostino & Pearson omnibus normality test. Unpaired *t*‐tests or Mann‐Whitney‐*U* test was used for cross sectional data; Wilcoxon matched pairs signed rank test for longitudinal samples. Correlations were determined using standard linear regression. Statistical significance was defined as *P* < 0.05.

## Results

### DMF treatment changes the composition and cytokine profile of T cells, monocytes and B cells

**Figure 1:**
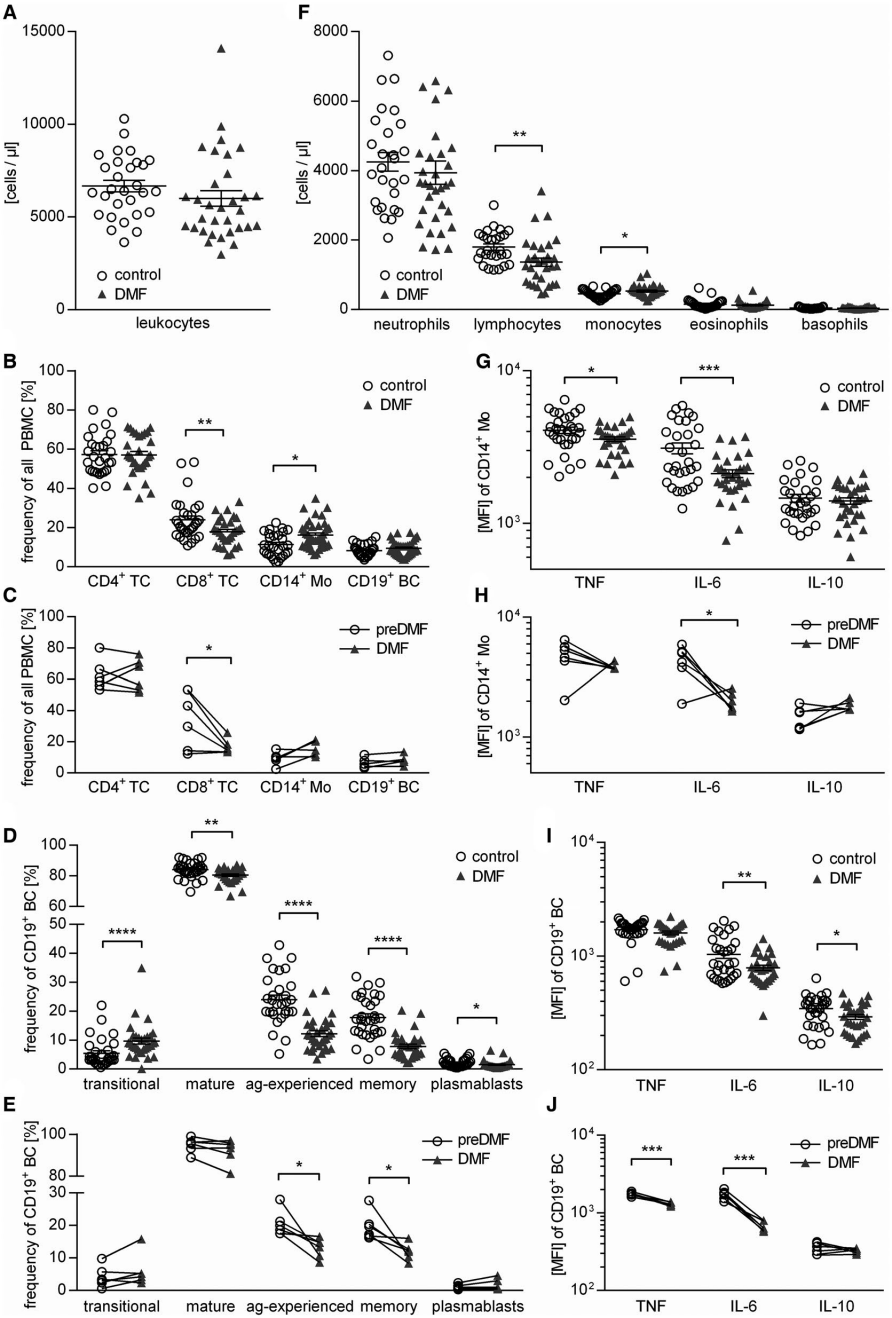
*DMF therapy changes cellular composition and cytokine production of human PBMC*. In a standard clinic’s blood evaluation, samples from dimethyl fumarate (DMF; n = 30) or non‐DMF (control; n = 31) treated multiple sclerosis patients were analyzed. Triangles represent DMF treatment, circles represent control treatment. (**A**) Total leukocyte count of the peripheral blood is presented (*ns*; unpaired t‐test). Peripheral blood mononuclear cells (PBMC) were isolated from the same patients; six multiple sclerosis patients were analyzed longitudinally upon DMF treatment. (**B**) Mean frequency ± standard error of the mean (SEM) of CD4^+^ T cells (TC), CD8^+^ TC, CD19^+^ B cells (BC) and CD14^+^ monocytes (Mo; *P < 0.05; **P < 0.01; unpaired *t*‐test). (**C**) Frequency of CD4^+^ TC, CD8^+^ TC, CD14^+^ Mo and CD19^+^ BC in PBMC of multiple sclerosis patients prior to and after DMF treatment initiation; line connects an individual patient (n = 6; *P < 0.05; Wilcoxon matched‐pairs signed rank test). (**D**) Mean frequency ± SEM of BC subpopulations defined as follows: Transitional BC (CD24^high^ CD38^high^; transitional), mature BC (CD24^var^ CD38^low^; mature), antigen‐experienced BC (CD27^+^; ag‐experienced), memory BC (CD27^var^ CD38^−^; memory) and plasmablasts (CD20^−^ CD27^+ ^CD38^+^; *P < 0.05; **P < 0.01; ****P < 0.0001; unpaired *t*‐test). (**E**) Frequency of transitional, mature, ag‐experienced and memory BC as well as plasmablasts in PBMC of multiple sclerosis patients prior to and after DMF treatment initiation; line connects an individual patient (n = 6; *P < 0.05; Wilcoxon matched‐pairs signed rank test). (**F**) Differential leukocyte counts of the peripheral blood are displayed (*P < 0.05; **P < 0.01; unpaired *t*‐test). (**G‐J**) After 20 h of pre‐incubation with 1 µg/mL CpG, PBMC were stimulated with 500 ng/mL ionomycin and 20 ng/mL phorbol 12‐myristate 13‐acetate for 4 h in the presence of a Golgi inhibitor and subsequently stained intracellularly for tumor necrosis factor (TNF), interleukin (IL)‐6 and IL‐10. (**G**) Shown is the respective mean fluorescence intensity (MFI) ± SEM of CD14^+^ Mo (*P < 0.05; ***P < 0.001; unpaired *t*‐test). (**H**) Cytokine production of CD14^+^ Mo in PBMC of multiple sclerosis patients prior to and after DMF treatment initiation shown as MFI; line connects an individual patient (n = 6; *P < 0.05; Wilcoxon matched‐pairs signed rank test). (**I**) Shown is the respective MFI ± SEM of CD19^+^ BC (*P < 0.05; **P < 0.01; unpaired *t*‐test). (**J**) Cytokine production of CD19^+^ BC in PBMC of multiple sclerosis patients prior to and after DMF treatment initiation shown as MFI; line connects an individual patient. (n = 6; ***P < 0.001; Wilcoxon matched‐pairs signed rank test).

At first, we analyzed the composition of PBMC in a MS patient cohort continuously receiving DMF and compared these findings to age‐ and sex‐matched MS patients who had never received DMF. Blood samples of six additional patients were analyzed prior to and after DMF treatment initiation (for epidemiological data see Table 1). In our standard clinic’s blood evaluation, DMF treatment induced an absolute reduction of lymphocytes and an increase in blood monocytes (Figure 1A,F). Stored PBMCs of the identical patients were analyzed in greater detail by flow cytometry; this analysis revealed that DMF treatment reduced the frequency of CD8^+^ T cells both in the cross‐sectional comparison as well as longitudinally, while the frequency of CD4^+^ T cells and CD19^+^ B cells remained unchanged. The frequency of circulating CD14^+^ monocytes was increased upon DMF treatment in line with the assessment of absolute cell numbers (Figure 1B,C). To assess the possible functional impact of this observation, we determined the cytokine production of CD14^+^ cells and detected a significant reduction of pro‐inflammatory TNF and IL‐6, while the release of anti‐inflammatory IL‐10 was unaltered (Figure 1G,H).

To study DMF treatment effects on B cells in detail, B cell maturation stages were divided into transitional (CD24^high^CD38^high^), mature naive (CD24^var^CD38^low^), antigen‐experienced (CD27^+^) and memory B cells (CD27^var^CD38^−^), as well as plasmablasts (CD20^−^ CD27^+ ^CD38^+^) (for gating see Supplementary Figure S1). While we observed a lower frequency of all mature B cell subsets in patients treated with DMF, the percentage of immature transitional B cells was inversely increased (Figure 1D,E,G). DMF treatment was further associated with a pronounced reduction of B cell‐produced IL‐6, and to a lower extent IL‐10 (Figure 1F,I). In the longitudinal samples, this effect was even more pronounced as both TNF and IL‐6 were reduced upon DMF treatment, while their IL‐10 production remained unchanged (Figure 1J; for gating see Supplementary Figure S2).

### DMF treatment reduces B cell expression of molecules involved in activation and antigen presentation

**Figure 2:**
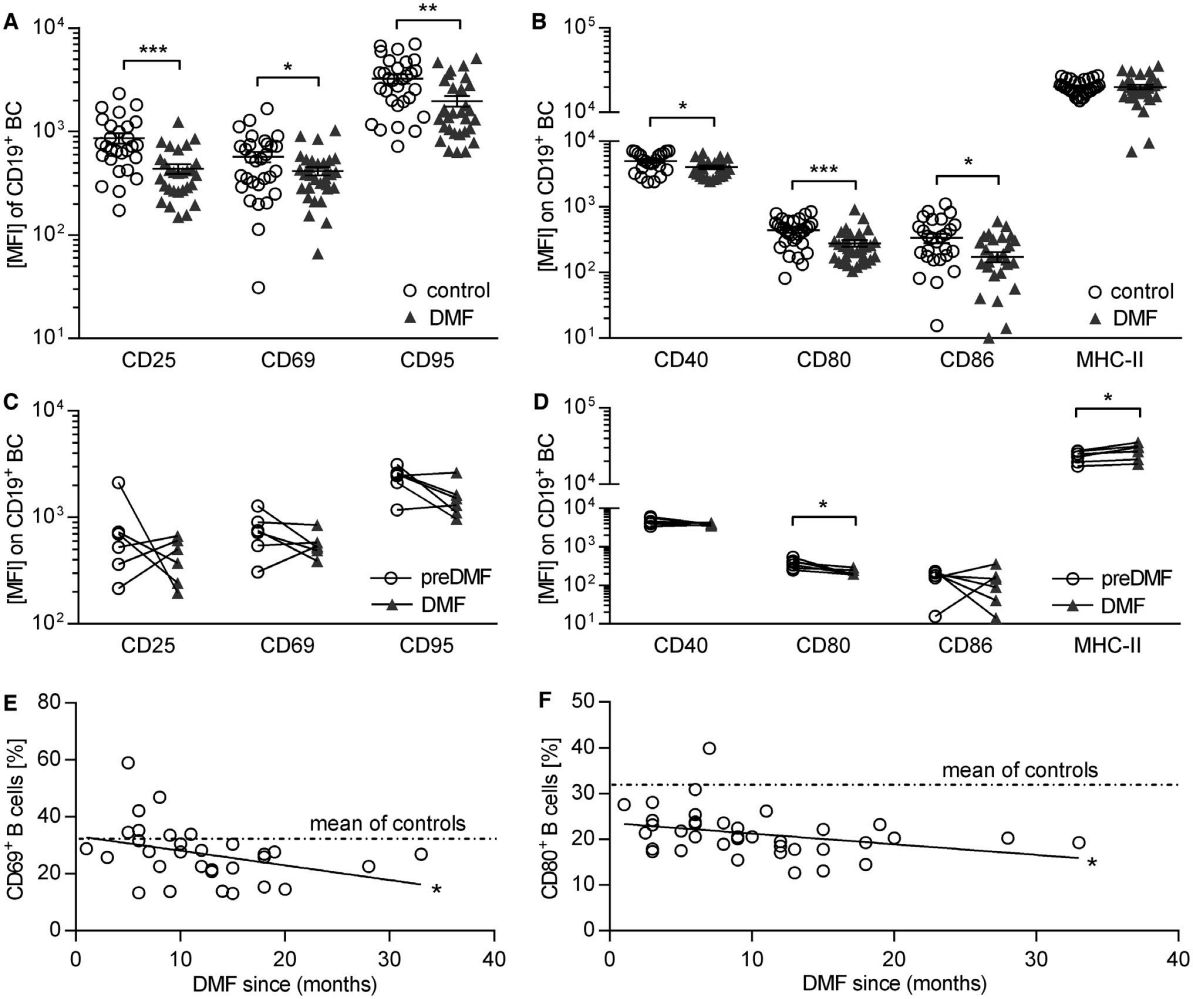
*Dimethyl fumarate treatment inhibits expression of activation markers and co‐stimulatory molecules on human B cells*. Peripheral blood mononuclear cells (PBMC) were isolated from dimethyl fumarate (DMF; n = 30) or non‐DMF (control; n = 31) treated multiple sclerosis patients; six multiple sclerosis patients were analyzed longitudinally upon DMF treatment. PBMC were incubated with 2 µg/mL for 20 h. Triangles represent DMF treatment, circles represent control treatment. (**A**) Mean fluorescent intensity (MFI) ± standard error of the mean (SEM) of activation markers expressed on B cells (BC; *P < 0.05; **P < 0.01; ***P < 0.001; unpaired *t*‐test) (**B**) MFI ± SEM of molecules on BC involved in antigen presentation (abbreviation: MHC class II = MHC‐II; *P < 0.05; ***P < 0.001; unpaired *t*‐test). (**C**) MFI of activation markers expressed on BC of multiple sclerosis patients prior to and after DMF treatment initiation; line connects an individual patient (n = 6; ns; Wilcoxon matched‐pairs signed rank test). (**D**) MFI of molecules on BC involved in antigen presentation of multiple sclerosis patients prior to and after DMF treatment initiation; line connects an individual patient (n = 6; *P < 0.05; Wilcoxon matched‐pairs signed rank test). (**E**) The individual patients’ frequencies of CD69^+^ BC (circles) were correlated with the duration of DMF treatment indicated by the Pearson correlation coefficient (*r* = 0.3654; *P *= 0.0433 solid line; *P < 0.05). The dashed lines represent the mean value of non‐DMF treated multiple sclerosis patients (mean of controls). (**F**) The individual patients’ frequencies of CD80^+^ BC (circles) was correlated with the duration of DMF treatment indicated by the Pearson correlation coefficient (*r* = 0.2356; *P* = 0.1381; ns). The dashed lines represent the mean value of non‐DMF treated multiple sclerosis patients (mean of controls).

Next, we analyzed the effect of DMF on B cell activation and their molecular capacity to act as APC. DMF treatment significantly diminished the expression of all analyzed activation markers (Figure 2A,C) and molecules involved in antigen presentation in the cross‐sectional cohort, while the expression of MHC‐II remained unchanged (Figure 2B,D). Moreover, we observed a continuous decline of CD69 and CD80 expression over time (Figure 2E,F) suggesting that DMF dampens the pro‐inflammatory B cell activity. Of note, these changes were solely associated with DMF treatment as we found no correlation with patient age, gender, expanded disability status scale score, disease duration or pre‐treatment in all study arms (Supplementary Figure S3–S8).

### DMF treatment ameliorates disease severity in an inflammatory experimental model actively involving B cells

**Figure 3:**
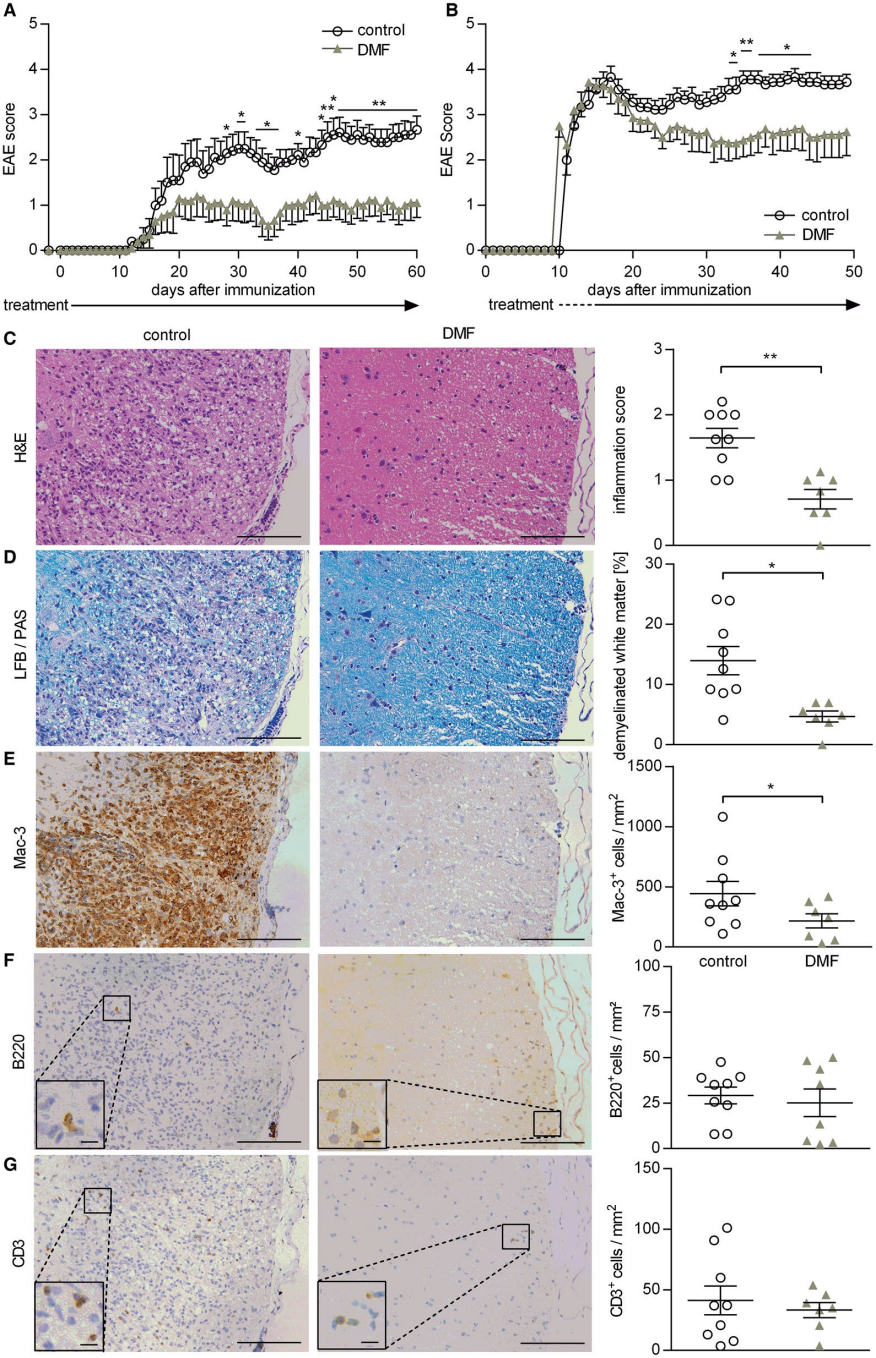
*Preventive and therapeutic dimethyl fumarate treatment ameliorates clinical and histological experimental autoimmune encephalomyelitis*. (**A**) C57BL/6 mice were immunized with MOG protein_1‐117_ and treated with 15 mg/kg dimethyl fumarate (DMF) or vehicle (control) twice a day (d) from d −2 until d 60 post immunization (p.i.). Mean group experimental autoimmune encephalomyelitis (EAE) score ± standard error of the mean (SEM; n = 10 mice/group; *P < 0.05; **P < 0.01; daily comparisons via Mann‐Whitney *U* test; data represent three independent experiments). (**B**) C57BL/6 mice were immunized with MOG protein_1‐117_ and therapeutically treated with 15 mg/kg DMF or control twice a day until d50 p.i. Therapeutic DMF treatment started when mice showed a score of 2 (hind limb weakness) or higher. Mean group EAE score ± SEM (n = 8–9 mice/group; *P < 0.05; **P < 0.01; daily comparisons via Mann‐Whitney *U* test; data represent three independent experiments). (**C–G**) C57BL/6 mice were immunized with MOG protein_1‐117_ and therapeutically treated with 15 mg/kg DMF or control twice a day until d50 p.i. Therapeutic DMF treatment started when mice showed a score of 2 (hind limb weakness) or higher. Inflammation (H&E; C), demyelination (LFB/PAS; D) and immune cell infiltration of the spinal cord evaluated by Mac‐3‐ B220‐ and CD3‐immunohistochemistry (**E–G**). Representative sections of controls (left) vs. therapeutically treated mice (middle) are shown. The mean spinal cord inflammation score ± SEM (0 = no inflammation, 1 = slight inflammation, 2 = moderate inflammation, 3 = strong inflammation), mean percentage of demyelinated white matter area and the mean number of infiltrating immune cells/mm^2^ are illustrated on the right (*P < 0.05; **P < 0.01; unpaired *t*‐test); scale bar = 100 μm (large pictures)/7.5 µm (inserts).

To elucidate the functional impact of the alterations observed in blood, the only accessible compartment in patients, we utilized an EAE model, in which both T and B cells contribute to disease pathogenesis. For this purpose, mice were immunized with natively folded MOG protein_1‐117_. As shown in Figure 3A, DMF treatment commenced prior to EAE induction prevented development of severe EAE and stabilized mice on a lower severity level. Importantly, this ameliorating effect was also seen when treatment started at the peak of disease, when clinical symptoms were fully established (Figure 3B). In both settings, the serum levels of anti‐MOG antibodies were not affected by DMF treatment (Supplementary Figure S9). However, histological analysis of these mice revealed a corresponding significant amelioration of CNS inflammation (Figure 3C) and demyelination (Figure 3D) accompanied by a reduction of activated Mac‐3^+^ cells within the CNS. However, at this late stage, the number of CNS‐infiltrating T and B cells showed only a trend to a decline (Figure 3E–G).

### DMF treatment abolishes development of encephalitogenic T cells in lymphoid organs

**Figure 4:**
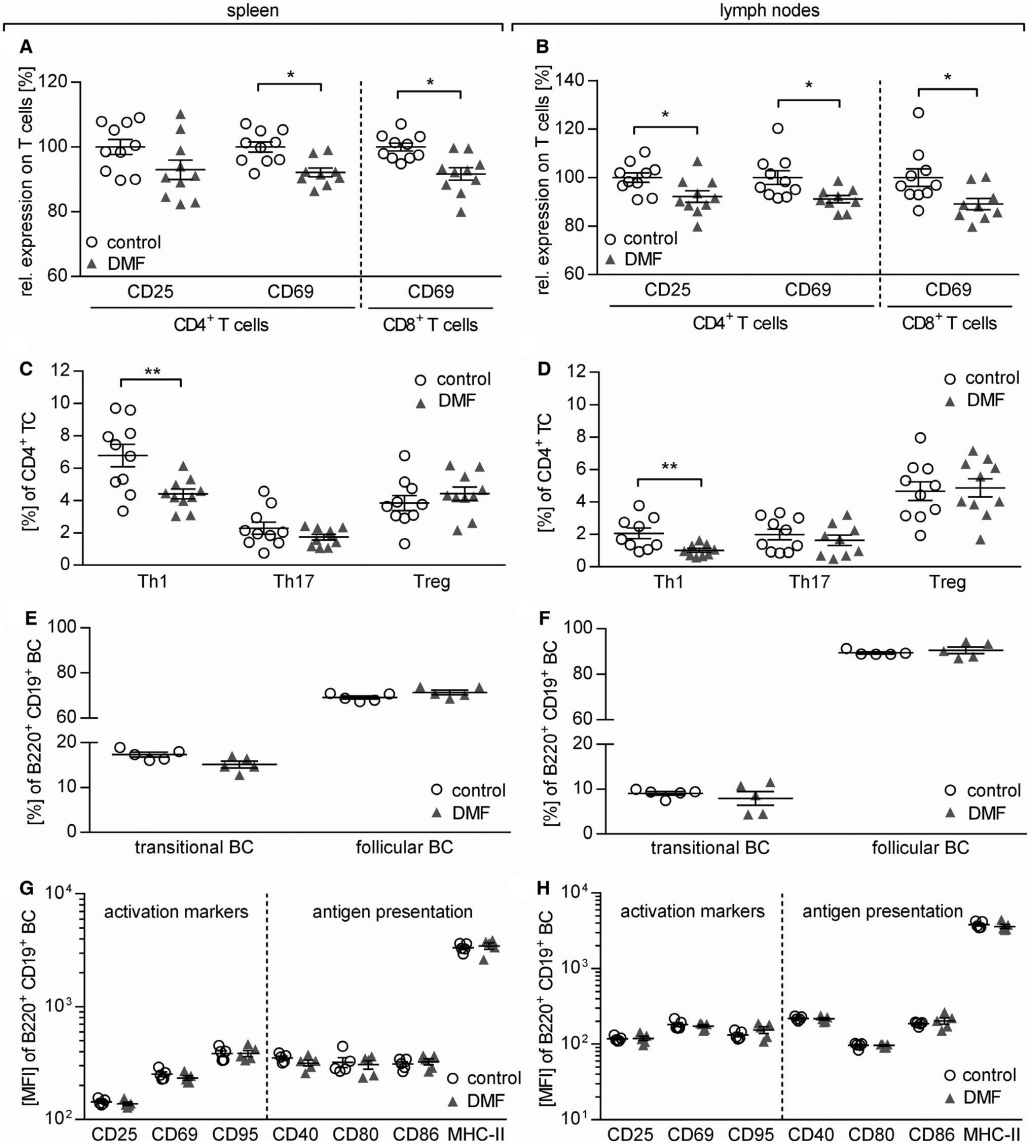
*Dimethyl fumarate treatment reduces T cell, but not B cell activation and differentiation in secondary lymphoid organs*. (**A+B**) Mice were immunized with MOG protein_1‐117_ and treated with 15 mg/kg dimethyl fumarate (DMF) or control twice a day from day (d)7 until d12 post immunization (p.i). The expression of CD25 and CD69 on (**A**) splenic and (**B**) lymph node CD4^+^ and CD8^+^ T cells (TC) normalized to the mean of controls (set to 100%) is shown as mean frequency ± standard error of the mean (SEM) and represent two independent experiments (*P < 0.05; **P < 0.01; unpaired *t*‐test). (**C,D**) Mean frequency ± SEM of splenic Th1 (IFN‐γ^+^), Th17 (IL17^+^) and regulatory (Treg; Foxp3^+^/CD25^+^) TC in (**C**) spleen and (**D**) lymph nodes. Data represent two independent experiments (**P < 0.01; unpaired *t*‐test). (**E,F**) Mean frequency ± SEM of transitional (CD21^hi^ CD23^+^) and follicular B cells (BC; CD21^int^ CD23) in spleen (*P < 0.05; unpaired *t*‐test) and lymph nodes (ns; unpaired *t*‐test). (**G,H**) Mean fluorescent intensity (MFI) ± SEM of activation markers (CD25, CD69 and CD95) and molecules involved in antigen presentation (CD40, CD80 and CD86 and MHC–II) on (**G**) splenic and (**H**) lymph node BC (B220^+^ CD19^+^) analyzed *ex vivo* by flow cytometry (ns; *P < 0.05; unpaired *t*‐test).

To dissect on the cellular level which immunological mechanisms contribute to this clinical benefit, we analyzed T and B cells in blood, lymph node and spleen of mice with early EAE, 12 days after immunization. The mean frequency of CD4^+^ T cells in the blood was reduced from 14.7% (±1.8%) in control‐treated to 12.6% (±1.5%) in DMF‐treated mice (*P* = 0.0289), while the frequency was increased from 20.0% (±2.7%) to 23.2% (±1.0%) in the lymph nodes (*P* = 0.0242) and from 12.8% (±2.4%) to 15.4% (±1.9%) in the spleen (*P* = 0.0157). Further, the frequency of CD8^+^ T cells was increased from 7.1% (±1.8%) in control‐treated to 8.9% (±1.3%) in DMF‐treated mice (*P* = 0.0178; all Mann Whitney *U* test; Supplementary Table S1). Next, we evaluated the activation, maturation and differentiation of these T cells in this early EAE priming phase. DMF treatment reduced the expression of CD25 on CD4^+^ T cells and of CD69 on both CD4^+^ and CD8^+^ T cells in spleen and lymph nodes (Figure 4A,B). Furthermore, DMF treatment diminished differentiation of Th1 cells in both the spleen and lymph node, while the frequencies of Th17 and regulatory T cells remained unaltered (Figure 4C,D). Next, we examined the expression of CD44, a marker T cells acquire upon antigen‐mediated activation 1. As depicted in Supplementary Figure S10, DMF‐treated mice revealed a lower frequency of highly activated CD4^+^ and CD8^+^ T cells in the spleen.

### DMF dampens the capacity of B cells to act as antigen‐presenting cells for the activation of T cells

Based on the altered phenotype of B cells in the blood of DMF‐treated patients, we next investigated whether DMF also influences B cell properties in secondary lymphoid organs of mice and to what extent such changes may reduce the development of effector T cells. We observed no overall reduction in the frequency of B cells in blood (*P* = 0.4336), lymph nodes (*P* = 0.0729) and spleen (*P* = 0.9002) of mice corresponding to the blood findings in patients. Also the frequency of CD21^int^ CD23^+^ follicular B cells and CD21^hi^ CD23^+^ transitional B cells in spleen and the lymph nodes of DMF‐treated mice remained unaltered (Figure 4E,F). Next, we analyzed the influence of DMF on B cell activation by assessing CD25, CD69 and CD95 and their molecular capacity to act as APC by quantifying CD40, CD80, CD86 as well as MHC‐II in spleen and lymph nodes. At this early time‐point, no significant changes in the expression of these molecules could be visualized (Figure 4G,H).

**Figure 5:**
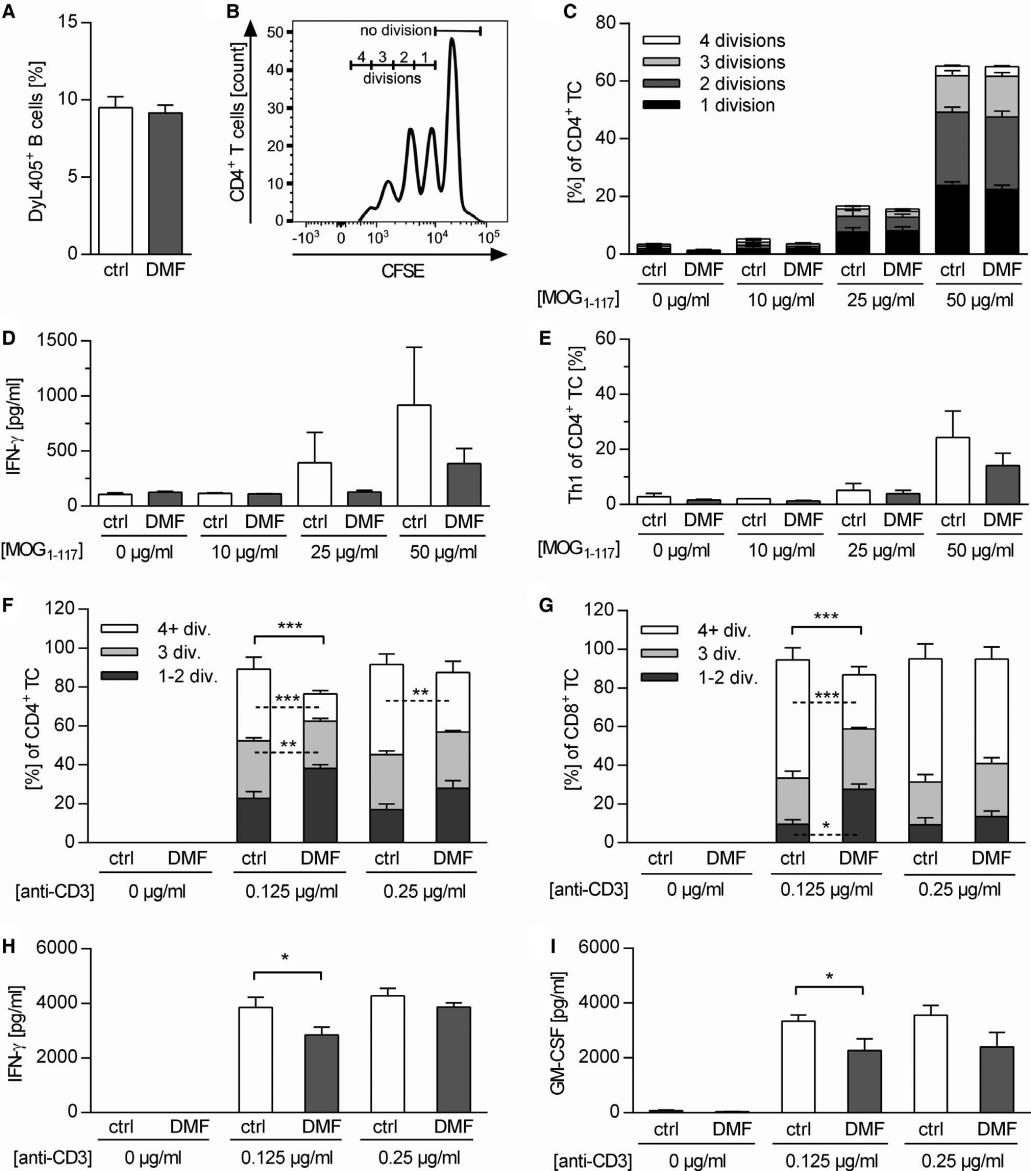
In vivo *dimethyl fumarate‐treated B cells do not inhibit T cell proliferation, but their activation*. (**A**) C57BL/6 mice were immunized with MOG protein_1‐117_ (rMOG_1‐117_) and treated with 15 mg/kg dimethyl fumarate (DMF) or vehicle (control) twice a day (d) from d‐7 until d12 post immunization (p.i.). Thereafter, splenic B cells (BC) were purified and incubated *in vitro* with fluorescence‐labeled MOG protein_1‐117_ for 2 h. Frequency of MOG‐binding BC (DyL405^+^) was evaluated by flow cytometry as mean frequency ± standard error of the mean (SEM; ns; unpaired *t*‐test). (**B,C**) BC were purified from mice immunized with rMOG_1‐117_ and additionally treated with 15 mg/kg DMF or control twice a day from d2 until d12 p.i. These isolated BC were then co‐cultured with CFSE‐labeled 2D2 T cells (TC) and stimulated with 0, 25 or 50 µg/mL rMOG_1‐117_. TC proliferation was analyzed by flow cytometry and evaluated using the number of divisions. (**B**) Representative histograms of proliferating 2D2 TC co‐cultured with BC. (**C**) Mean frequency ± SEM of proliferating TC further classified by the number of divisions are shown (n = 5, ns; unpaired *t*‐test). (**D, E**) IFN‐γ production of CD4^+^ TC was analyzed by ELISA and Th1 cell frequency was assessed using intracellular flow cytometric staining at different concentrations of rMOG_1‐117_ (n = 4 mice/group; ns, unpaired *t*‐test). (**F–I**) Naïve C57BL/6 mice were treated with 15 mg/kg DMF or control twice a day for 19 days. Purified CFSE‐labeled (**F**) CD4^+^ and (**G**) CD8^+^ TC were stimulated *in vitro* with 0, 0.125 or 0.25 µg/mL anti‐CD3 and 5 µg/mL anti‐CD28 antibodies. Mean frequency ± SEM of proliferating TC additionally classified by divisions (div.) are shown (n = 4 mice/group; *P < 0.05, **P < 0.01, ***P < 0.001, unpaired *t*‐test). (**H**) IFN‐γ and (**I**) GM–CSF concentrations determined by ELISA are shown as mean ± SEM (*P < 0.05; unpaired *t*‐test).

Furthermore, we investigated how DMF exposure may alter the B cell ability to act as APC for the activation of T cells. First, we determined the capacity of *in vivo* DMF‐treated B cells to bind MOG protein_1‐117_, the immunogen used for EAE induction. The antigen‐binding capacity of the B cell receptor was not altered upon DMF treatment (Figure 5A), which concomitantly indicates that DMF did not prevent development of auto‐reactive B cells in this model. To directly assess their APC capacity, we co‐cultured B cells from immunized, DMF‐treated mice with naïve MOG peptide_35‐55_‐specific T cells in the presence of MOG protein_1‐117 _. Of note, the added T cells had never been treated with DMF. As indicated in Figure 5B and C, the proliferation of T cells activated by DMF‐treated B cells was indistinguishable from the control setting. However, significantly fewer CD4^+ ^T cells differentiated into Th1 cells when activated by DMF‐treated B cells (Figure 5D,E), while no effect was seen for the frequency of Th17 cells (Supplementary Figure S11).

In a complementary setting, naïve un‐immunized mice were treated with DMF and T cells were directly activated ex vivo by anti‐CD3 and anti‐CD28. As shown in Figure 5F and G, *in vivo* DMF exposure of T cells was associated with a lower proliferation rate as well as with a reduced release of IFN‐γ and GM‐CSF (Figure 5H,I). Taken together, these data suggest that the observed inhibition in the development of disease‐driving T cells upon DMF treatment is mediated by both a direct effect on T cells and a synergistic effect on B cells, weakening their APC capacity.

### In a model of toxic demyelination, DMF treatment protects oligodendrocytes and reduces axonal damage

**Figure 6:**
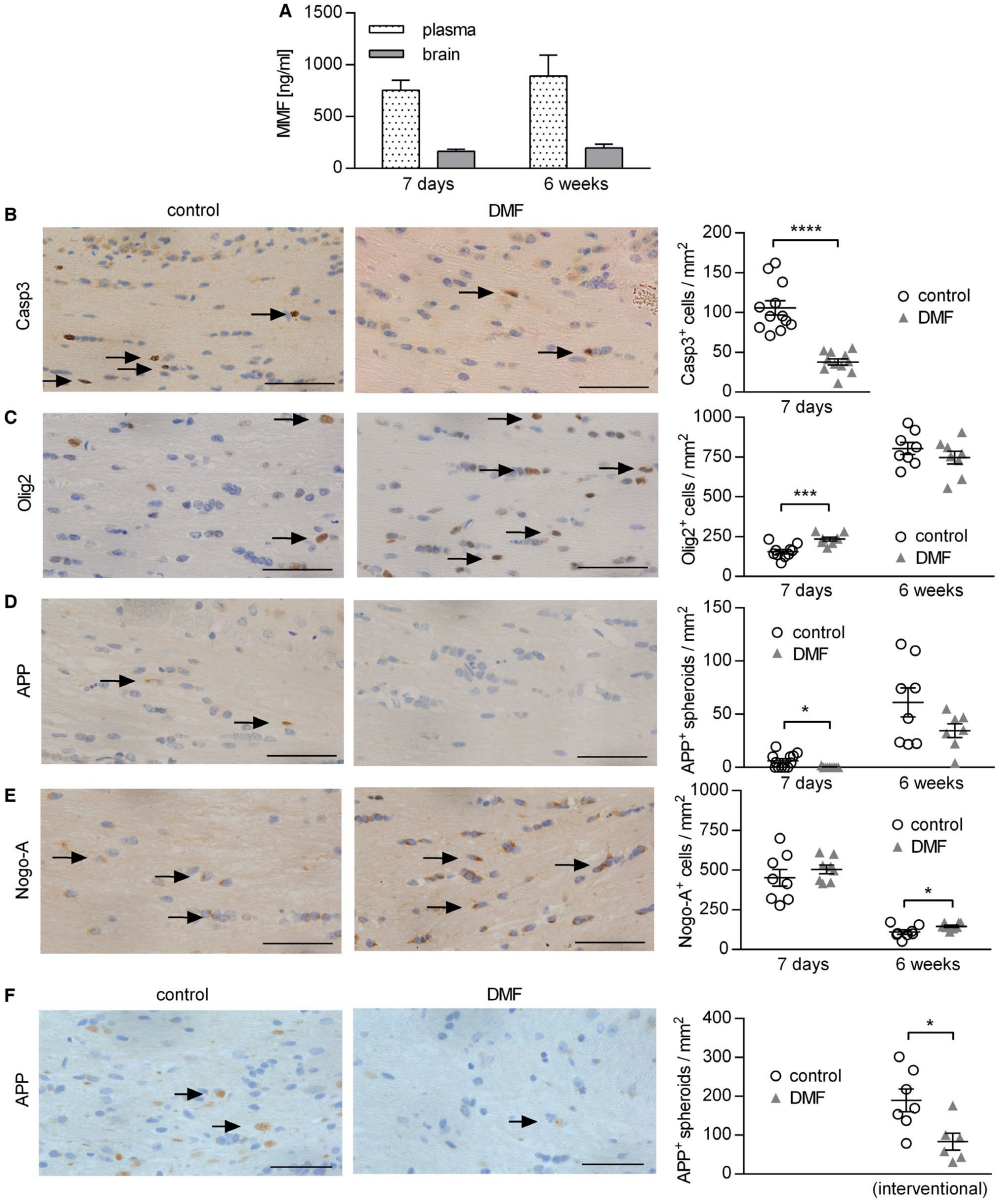
*Dimethyl fumarate treatment reduces cuprizone‐induced apoptosis of oligodendrocytes and acute axonal damage in the corpus callosum*. (**A**) C57BL/6 mice were treated with 15 mg/kg dimethyl fumarate (DMF) twice a day for 7 days or 6 weeks. The concentration of the DMF metabolite monomethyl fumarate (MMF) was analyzed by mass spectrometry in plasma and brain shown as mean ± standard error of the mean (SEM; n = 4–6 mice/time point). (**B–E**) C57BL/6 mice were fed with 0.25% cuprizone for 7 days or 6 weeks and simultaneously treated with 15 mg/kg DMF or vehicle (control) twice a day. Immunohistochemistry of brain sections was performed to determine the number of (**B**) apoptotic cells (caspase–3 (Casp3)^+^), (**C**) oligodendrocytes including progenitor cells (Olig2^+^), (**D**) acute axonal damage (amyloid precursor protein (APP)^+^ spheroids) and (**E**) mature oligodendrocytes (Nogo‐A^+^) in the corpus callosum. Representative sections of the corpus callosum after 7 days are shown on the left and quantifications of cells/mm^2^ on the right as mean ± SEM. (**F**) C57BL/6 mice were fed with 0.25% cuprizone for 5 weeks. Afterwards, cuprizone diet was stopped and mice were treated with 15 mg/kg DMF or control twice a day for 3 days, before immunohistochemical staining for acute axonal damage (APP^+^ spheroids) was performed. Representative sections of the corpus callosum and quantification of APP^+^ spheroids/mm^2^ as mean ± SEM are shown (n = 8–12 mice/time point; *P < 0.05; ***P < 0.001; ****P < 0.0001; unpaired *t*‐test); scale bar = 50 µm.

In the second part of our study, we investigated whether DMF exerts CNS intrinsic effects independent of its immunomodulatory properties. Therefore, we utilized the murine cuprizone model, a model of toxic demyelination characterized by apoptosis of oligodendrocytes, axonal damage and activation of CNS resident cells. In this model, the blood‐brain barrier remains intact and hematogenous immune cells do not contribute in a significant manner 41. First, we validated that the active metabolite of DMF, MMF, was detectable in plasma and brain samples of naïve mice treated for 7 days with DMF; its concentration even slightly increased upon long‐time therapy for 6 weeks (Figure 6A). To investigate the effect of DMF in a short‐term regimen, we administered it in parallel to cuprizone for 7 days and observed that DMF significantly reduced the number of caspase‐3^+^ apoptotic oligodendrocytes (Figure 6B). Treatment also resulted in higher numbers of Olig2^+^ oligodendrocytes, including oligodendrocyte precursor cells (OPC; Figure 6C). Besides this protective effect on oligodendrocytes, DMF almost completely abolished the acute axonal damage measured by the number of APP^+^ spheroids (Figure 6D). We next assessed the effect of DMF in a long‐time regimen in which cuprizone and DMF were administered for 6 weeks. Complementary to the short‐time results, DMF‐treated mice showed a significantly higher number of mature Nogo‐A^+^ oligodendrocytes in the brain, while at this late time point no difference in the number of Olig2^+^ oligodendrocytes was found (Figure 6C,E). A trend toward less acute axonal damage was observed in DMF‐treated animals (Figure 6D). Lastly, we investigated to what extent DMF may exert neuroprotective effects in an interventional setting in which the CNS damage had already occurred. Therefore, mice were fed with cuprizone for 5 weeks, then the cuprizone diet was replaced by normal chow and mice were treated with DMF or control. In this setting, no effect on the activation status of myeloid cells was observed (Supplementary Figure S12). While the extent of demyelination was comparable in both groups (Supplementary Figure S13), DMF treatment significantly decreased the number of APP^+^ spheroids (Figure 6F), indicating that therapeutic DMF treatment reduced the number of damaged axons even after toxic CNS damage had fully developed.

### DMF may exert neuroprotective effects in human CNS demyelinating disease

**Figure 7:**
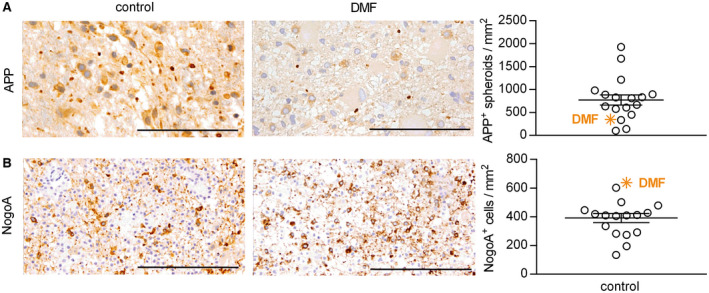
*In a brain biopsy from a dimethyl fumarate‐treated multiple sclerosis patient, axonal damage is reduced and the number of mature oligodendrocytes is increased when compared to a treatment‐naive multiple sclerosis biopsy cohort*. A brain biopsy from a patient with multiple sclerosis receiving dimethyl fumarate (DMF) treatment for 2 months (indicated by the orange symbol) was analyzed for the number of (**A**) acutely damaged axons (amyloid precursor protein (APP)^+^) and (**B**) mature oligodendrocytes (Nogo‐A^+^). Results were compared to brain lesions from patients that had not received any immunomodulatory or immunosuppressive therapy (control). Representative sections are shown on the left and quantification of APP^+^ and Nogo‐A^+^ cells/mm^2^ on the right as mean ± standard error of the mean; scale bar APP = 50 µm; scale bar Nogo‐A = 100 µm.

Based on these experimental findings, we took the opportunity to analyze one available brain biopsy of an acute lesion obtained from a DMF‐treated MS patient. We compared this single sample to a lesion stage‐matched CNS biopsy cohort of treatment‐naïve patients with MS (for epidemiological data see Table 1). In comparison to this control group, the CNS sample from the DMF‐treated MS patient revealed a lower number of acutely damaged axons, along with a comparably high number of Nogo‐A^+^ mature oligodendrocytes (Figure 7A,B).

## Discussion

We investigated the effect of DMF both on pathogenic B cell properties as well as on ongoing CNS damaging processes in mice and humans. We found that DMF treatment selectively impaired differentiated B cell stages both by decreasing the prevalence of these phenotypes and by directly inhibiting the expression of molecules with potentially pathogenic properties. In blood obtained from DMF‐treated MS patients, we observed a strong decline in mature and memory B cells as well as plasmablasts, whereas the frequency of immature, transitional B cells was elevated. These findings are in line with an overall shift from potentially pathogenic B cells phenotypes toward naïve, non‐pathogenic entities. Reflective of this assumption, we found a pronounced decrease in the production of pro‐inflammatory IL‐6 and TNF by B cells, supporting earlier reports of an anti‐inflammatory shift of B cell cytokines upon DMF treatment 31. Lastly, we observed a downregulation of CD25, CD69 and CD95, suggesting that the overall capacity of remaining B cells to react to stimulation was decreased. Interestingly, all these inhibitory immunological alterations intensified the longer an individual patient received DMF, suggesting that continuous treatment is required to promote these transitions.

Some of these findings appear not to be specific for B cells: We could show a similar decrease in the release of IL‐6 and TNF by monocytes from DMF‐treated patients, while their production of anti‐inflammatory IL‐10 tended to increase. Along the same lines, the identical activation markers found to be decreased on human B cells, CD25 and CD69, were reduced on murine T cells, which is concordant with a broad inhibitory effect of DMF on T cell activation and differentiation 15, 50. Taken together, these findings support the concept of a wide‐ranging effect of DMF on different immune cells. Its predominant influence on differentiated immune cell phenotypes may be explained by the most recently proposed hypothesis that MMF interferes with aerobic glycolysis and hereby affects all immune cells with a higher metabolic turnover 26.

For functional analyses of DMF effects on B cells, we utilized a murine EAE model, in which both B and T cells contribute in a pathogenic manner 49. DMF treatment initiated prior to disease induction as well as after EAE was fully established significantly reduced disease severity, CNS inflammation and demyelination. While this benefit was associated with a decline in the number of activated myeloid cells within the CNS, DMF had no significant effect on the prevalence of B and T cells at this late time‐point. Earlier adaptive immune responses were however strongly diminished in lymphoid organs. Paralleling our findings in human blood, DMF treatment reduced the activation of T cells in lymph nodes and spleen, resulting in a diminished differentiation into encephalitogenic effector cells. When activating T cells from DMF‐treated, un‐immunized mice directly *ex vivo*, we observed that *in vivo* DMF exposure had diminished their capacity to expand and differentiate in an encephalitogenic manner. Jointly, our findings support the concept that DMF exerts a direct inhibitory effect on T cells 11, 20. Analyzing B cells in lymphoid organs, we detected a tendency toward a more naïve, non‐activated phenotype. These alterations were clearly less impressive when compared to the effect of continuous DMF treatment on human blood B cells. This may indicate that continuous DMF‐mediated B cell immune modulation occurs steadily over time and thus increases in its extent beyond the treatment window of actively induced EAE. Nevertheless, functional analyses showed that splenic B cells from DMF‐treated mice dampened the generation of Th1 effector cells. Taken together, these findings establish that modulation of both T and B cells results in a synergistic impairment to generate disease‐driving effector T cells. Furthermore, the novel observation that DMF impairs frequency and function of differentiated B cells suggests that DMF may be a suitable medication to prevent re‐development of pathogenic B cell function after anti‐CD20 mediated B cell depletion.

One primarily unrelated, yet possibly meaningful observation derives from comparing frequencies of immune cells in blood, lymph node and spleen in DMF‐treated mice. Paralleling the findings in treated humans, we observed a decline in the frequency of T cells in the blood of mice. In contrast, the frequency of both CD4^+^ and CD8^+^ T cells in spleen and lymph node was significantly elevated in DMF‐treated mice, raising the question whether part of the detected blood T‐lymphocytopenia may refer to a shift of T cells into secondary lymphoid organs. Concomitantly, these findings challenge the concept that DMF and its metabolite MMF lowers blood abundancy of T cells exclusively by promoting apoptosis, which primarily occurs at high concentrations of fumarates 12. Hence, if this complementary perspective on low blood T cell counts evoked by a shift into lymphoid organs holds true, it may indicate that reversal of this (side) effect of DMF could occur faster and more thoroughly upon treatment cessation than previously thought 9.

Our histologic analysis of EAE mice showed that DMF treatment substantially lowered the abundance of activated myeloid cells in mice in which DMF treatment was initiated after disease induction. As myeloid cells in this analysis composed of infiltrated macrophages, but also of CNS resident microglia, we were interested in CNS intrinsic effects and utilized the cuprizone model of toxic demyelination in which CNS damage occurs largely independent of the peripheral immune system. We first demonstrated that MMF reaches the CNS, which of course is an important precondition for any direct effect within the CNS. Although a prior study using the cuprizone model could not find clear neuroprotective effects 40, we showed that DMF reduced the apoptosis of oligodendrocytes, preserved a higher number of Olig2^+^ oligodendrocytes at early stages and increased the number of mature oligodendrocytes after 6 weeks. These results suggest protective properties on oligodendrocytes and possibly indicate promotion of oligodendrocyte differentiation, which could be of enormous therapeutic importance, since in MS lesions OPC are present, but fail to mature 28. Although an indirect effect on oligodendrocytes by infiltrating cells cannot be excluded with certainty 23, 33, 37, the beneficial effects on oligodendrocytes may be explained by *in vitro* findings that provide evidence for direct oligodendrocyte protective effects: DMF treatment increases the levels of antioxidant molecules including carnitine, ascorbic acid as well as glutathione in human oligodendrocytes 21, and reduces oligodendrocyte apoptosis induced by oxidative stress 51. In addition, by binding Kelch‐like ECH‐associated protein 1, DMF is thought to promote the translocation of Nrf2 into the nucleus elevating the expression of several detoxification enzymes 32, 38. Indicative of this assumption was our observation that the nuclear expression of Nrf2 is upregulated in glial cells of DMF‐treated MS patients 38. DMF‐mediated Nrf2 activation is also thought to lead to a better protection of axons, as shown in a model of experimental autoimmune neuritis 43. In MS, acute axonal damage mainly occurs during the early stages of disease and correlates with the extent of inflammation 27. In our model, we could show that DMF significantly reduces the number of acutely damaged axons. Interestingly, this beneficial effect was also seen when DMF was applied for 3 days after cuprizone withdrawal, suggesting an early neuroprotective effect of therapeutic DMF application, which may explain the accentuated effect of early DMF treatment on brain atrophy in MS patients 7, 25.

Analyzing one available CNS biopsy of an acute lesion from a DMF‐treated MS patient, we could somewhat confirm a relative reduction of axonal damage and an increase in the number of mature oligodendrocytes. Unfortunately, this was the only DMF‐treated biopsy we could obtain, and we accordingly compared our findings to a treatment‐naïve MS biopsy cohort. While the analysis is thus far from being conclusive, it is generally concordant with a direct CNS protective effect of DMF and/or MMF as oligodendrocyte death and axonal injury are assumed major contributors to brain atrophy 4, 22 and long‐term disability in MS patients 8, 14, 16. From a clinical perspective, these novel findings both in mice and humans thus implicate that apart from its effect on the immune system and occurrence of relapses, DMF may limit the functional impairment resulting from an acute flare and decelerate transition to secondary progressive MS by restricting the extent of CNS neurodegeneration.

## Statement of Author Contribution

MW and IM conceived and directed the project. JT and ST carried out experiments, interpreted results and wrote the manuscript. JO, SS and RS carried out experiments. WB, SH contributed to interpretation of results and provided feedback on the manuscript. All authors had final approval of the submitted version.

## Conflict of Interest

The authors declare no competing financial interests. This study was funded by Biogen‐Idec.

## Supporting information


**Figure S1.** *General pre‐gating strategy and gating for B cell subsets and surface molecule expression.* (**A**) Within all recorded events, doublets were excluded and living cells were determined using size exclusion and staining with Zombie‐dye. (**B**) Within the living cell population (see A), B cells were defined as CD19^+^. Surface marker were evaluated using the MFI. B cell subpopulations were identified as follows: Antigen‐ (Ag‐) experienced B cells (CD27^+^), memory B cells (CD27^var^ CD38^‐^), plasmablasts (CD20^‐^ CD27^+^ CD38^+^), mature B cells (CD24^var^ CD38^low^) and transitional B cells (CD24^high^ CD38^high^).
**Figure S2.** *Gating strategy for intracellular cytokine staining in B cells and monocytes.* After 20 hours of pre‐incubation with 1 μg/ml CpG, PBMC were stimulated with 500 ng/ml ionomycin and 20 ng/ml phorbol 12‐myristate 13‐acetate for 4 hours in the presence of a Golgi inhibitor and subsequently stained intracellularly for TNF, IL‐6 and IL‐10. **(A) **Within the living cell population (as defined in supplementary figure 1), B cells were defined as CD19^+^. Their cytokine production was quantified using the mean fluorescence intensity (MFI) of the respective fluorescence labeled cytokine antibody (TNF ‐ A700, IL‐6 ‐ FITC, IL‐10 ‐ PE‐CF594). **(B)** Within the living cell population (as defined in supplementary figure 1), monocytes were defined as CD14^+^. Their cytokine production was quantified using the mean fluorescence intensity (MFI) of the respective fluorescence labeled cytokine antibody (TNF ‐ A700, IL‐6 ‐ FITC, IL‐10 ‐ PE‐CF594).
**Figure S3.** *Correlation between cellular composition of human peripheral blood mononuclear cells and patient related data.* Immune cell frequencies in peripheral blood mononuclear cells of dimethyl fumarate treated (DMF; triangle) or control (circle) multiple sclerosis patients were correlated to **(A)** patient age, gender and expanded disability status scale (EDSS) score as well as **(B)** disease duration, premedication (interferon (IFN), glatiramer acetate (GA), Natalizumab (Nat), fingolimod (FTY)) and treatment duration using linear regression (solid line; * = *p *< 0.05). Bars indicate mean ± standard error of the mean. BC = B cells, Mo = monocytes.
**Figure S4.** *Correlation between B cell subpopulations and patient related data.* Transitional BC (CD24^high^ CD38^high^), mature BC (CD24^var^ CD38^low^), antigen‐experienced BC (CD27^+^; Ag‐exp.), memory BC (CD27^var^ CD38^‐^) and plasmablasts (CD20^‐^ CD27^+^ CD38^+^) were analyzed. B cell subpopulation frequencies of dimethyl fumarate treated (DMF; triangle) or control (circle) patients were correlated to **(A)** patient age, gender and expanded disability status scale (EDSS) score as well as **(B)** disease duration, premedication (interferon (IFN), glatiramer acetate (GA), Natalizumab (Nat), fingolimod (FTY)) and treatment duration using linear regression (solid line; * = *p *< 0.05). Bars indicate mean ± standard error of the mean. BC = B cells.
**Figure S5.** *Correlation between B cell activation marker and patient‐related data.* Peripheral blood mononuclear cells were stimulated with 2μg/ml CpG for 20 hours. The expression of B cell activation marker (evaluated as mean fluorescent intensity: MFI) of dimethyl fumarate treated (DMF; triangle) or control (circle) patients were correlated to **(A)** patient age, gender and expanded disability status scale (EDSS) score as well as **(B)** disease duration, premedication (interferon (IFN), glatiramer acetate (GA), Natalizumab (Nat), fingolimod (FTY)) and treatment duration using linear regression (solid line; * = *p *< 0.05). Bars indicate mean ± standard error of the mean.
**Figure S6.** *Correlation between antigen presentation‐related B cell marker and patient related data.* Peripheral blood mononuclear cells were stimulated with 2μg/ml CpG for 20 hours. The expression of antigen presentation‐related B cell marker (evaluated as mean fluorescent intensity: MFI) of dimethyl fumarate treated (DMF; triangle) or control (circle) patients were correlated to **(A)** patient age, gender and expanded disability status scale (EDSS) score as well as **(B)** disease duration, premedication (interferon (IFN), glatiramer acetate (GA), Natalizumab (Nat), fingolimod (FTY)) and treatment duration using linear regression (solid line; * = *p *< 0.05). Bars indicate mean ± standard error of the mean.
**Figure S7.** *Correlation between B cell‐produced cytokines and patient related data.* After 20 hours of pre‐incubation with 1 μg/ml CpG, peripheral blood mononuclear cells were stimulated with 500 ng/ml ionomycin and 20 ng/ml phorbol 12‐myristate 13‐acetate for 4 hours in the presence of a Golgi inhibitor and subsequently stained intracellularly for TNF, IL‐6 and IL‐10. Cytokines produced by CD19^+^ B cells (evaluated as mean fluorescent intensity: MFI) of dimethyl fumarate treated (DMF; triangle) or control (circle) patients were correlated to **(A)** patient age, gender and expanded disability status scale (EDSS) score as well as **(B)** disease duration, premedication (interferon (IFN), glatiramer acetate (GA), Natalizumab (Nat), fingolimod (FTY)) and treatment duration using linear regression (solid line; * = *p *< 0.05). Bars indicate mean ± standard error of the mean.
**Figure S8.** *Correlation between monocyte‐produced cytokines and patient related data.* After 20 hours of pre‐incubation with 1 μg/ml CpG, peripheral blood mononuclear cells were stimulated with 500 ng/ml ionomycin and 20 ng/ml phorbol 12‐myristate 13‐acetate for 4 hours in the presence of a Golgi inhibitor and subsequently stained intracellularly for TNF, IL‐6 and IL‐10. Cytokines produced by CD14^+^ monocytes (evaluated as mean fluorescent intensity: MFI) of dimethyl fumarate treated (DMF; triangle) or control (circle) patients were correlated to **(A)** patient age, gender and expanded disability status scale (EDSS) score as well as **(B)** disease duration, premedication (interferon (IFN), glatiramer acetate (GA), Natalizumab (Nat), fingolimod (FTY)) and treatment duration using linear regression (solid line; * = *p *< 0.05). Bars indicate mean ± standard error of the mean.
**Figure S9.** *Preventive and therapeutic dimethyl fumarate treatment do not alter anti‐MOG antibody levels in experimental autoimmune encephalomyelitis.* (**A**) C57BL/6 mice were immunized with MOG protein_1‐117_ and treated with 15 mg/kg dimethyl fumarate (DMF) or vehicle (control) twice a day (d) from d ‐2 until d 60 post immunization (p.i.). Mean anti‐MOG antibody levels in the serum ± standard error of the mean (SEM; *n *= 10 mice / group; *ns*; Mann‐Whitney U test; data represent three independent experiments). (**B**) Therapeutic DMF treatment started when mice showed a score of 2 (hind limb weakness) or higher. Mean anti‐MOG antibody levels in the serum ± standard error of the mean (SEM; *n *= 8‐9 mice / group; *ns*; Mann‐Whitney U test; data represent three independent experiments).
**Figure S10.** *DMF treatment reduces T cell differentiation in the spleen.* Mice were immunized with MOG protein_1‐117_ and treated with 15 mg/kg DMF or control twice a day from day (d)7 until d12 post immunization. **(A, B)** Representative dot plots of CD44 expression on CD4^+^ T cells in spleen and lymph nodes. Frequency ± standard error of the mean of **(C)** splenic and **(D)** lymph node CD4^+^ and CD8^+^ T cells expressing high (CD44^hi^), intermediate (CD44^int^) and low (CD44^low^) levels of CD44 (* = *p* < 0.05; unpaired t‐test).
**Figure S11.** *In vivo DMF‐treated B cells do not alter Th17 differentiation.* B cells were purified from mice immunized with MOG protein_1‐117_ and additionally treated with 15 mg/kg dimethyl fumarate (DMF) or control twice a day (d) from d2 until d12 post immunization. These isolated B cells were then co‐cultured with CFSE‐labeled 2D2 T cells and stimulated with 0, 25 or 50 μg/ml MOG protein_1‐117_ (rMOG_1‐117_). T cell proliferation was analyzed by flow cytometry and evaluated using the number of divisions. **(A, B)** IL‐17 production of CD4^+^ TC was analyzed by enzyme‐linked immunosorbent assay and Th17 frequency was determined using intracellular flow cytometric staining at different concentrations of rMOG_1‐117_. (*n *= 4 mice / group; * = *p* < 0.05, ** = *p* < 0.01, *** = *p* < 0.001; unpaired t‐test).
**Figure S12.** *DMF treatment did not alter macrophage infiltration after long‐term cuprizone diet*. C57BL/6 mice were fed with 0.25% cuprizone and treated with 15 mg/kg DMF or vehicle (control) twice a day for six weeks. **(A)** Hematoxylin and eosin stain of the corpus callosum is presented. **(B)** Mac‐3^+^ cells in the corpus callosum after seven days (left graph) and six weeks (right graph) of DMF/control treatment in the cuprizone model (mean ± SEM; ns; unpaired t‐test).
**Figure S13.** *Demyelination of the corpus callosum was not majorly altered in preventative DMF treatment after long‐term cuprizone diet.* **(A)** C57BL/6 mice were fed with 0.25% cuprizone and treated with 15 mg/kg DMF or vehicle (control) twice a day for six weeks in a preventive setting. LFB/PAS and PLP staining was performed to determine the percentage of demyelinated area of the corpus callosum shown as mean ± SEM (ns; unpaired t‐test). **(B)** In an interventional setting, C57BL/6 mice were fed with 0.25% cuprizone for five weeks. Afterwards, cuprizone diet was stopped and mice were treated with 15 mg/kg DMF or control twice a day for three days. LFB/PAS staining was performed to determine the percentage of demyelinated area of the corpus callosum shown as mean ± SEM (ns; unpaired t‐test).
**Figure S14.** *DMF treatment did not change absolute leukocyte counts in different organs.* Mice were immunized with MOG protein_1‐117_ and treated with 15 mg/kg DMF or control twice a day from day (d)7 until d12 post immunization **(A)** Absolute leukocyte counts of the blood, the spleen, the lymph nodes and the thymus were determined after two weeks of DMF/control treatment. **(B)** Absolute leukocyte counts of the blood, the spleen, the lymph nodes and the thymus were determined after three weeks of DMF/control treatment (mean ± SEM; ns; unpaired t‐test).
**Table S1.** *Immune cell frequencies in blood (A), spleen (B) and lymph nodes (C) of mice are altered upon dimethyl fumarate treatment.* C57BL/6 mice were immunized with MOG protein_1‐117_ and treated with 15 mg/kg dimethyl fumarate DMF or vehicle (control) twice a day (d) from d ‐7 until d12 post immunization. Within all leukocytes, mean cell frequencies of CD4^+^ T cells, CD8^+^ T cells, CD19^+^ B cells and CD11b^+^ myeloid cells are shown. Data represent two independent experiments (* = *p* < 0.05; unpaired t‐test).
