# Supplementary material for: Dimethyl fumarate impairs differentiated B cells and fosters central nervous system integrity in treatment of multiple sclerosis
Source: Brain Pathol. 2019 Mar 5;29(5):640–57. doi: 10.1111/bpa.12711 (PMC6849574; doi:10.1111/bpa.12711)
Supplement: Supplementary file 1 — Figure S1. General pre‐gating strategy and gating for B cell subsets and surface molecule expression. (A) Within all recorded events, doublets were excluded and living cells were determined using size exclusion and staining with Zombie‐dye. (B) Within the living cell population (see A), B cells were defined as CD19+. Surface marker were evaluated using the MFI. B cell subpopulations were identified as follows: Antigen‐ (Ag‐) experienced B cells (CD27+), memory B cells (CD27var CD38‐), plasmablasts (CD20‐ CD27+ CD38+), mature B cells (CD24var CD38low) and transitional B cells (CD24high CD38high). Figure S2. Gating strategy for intracellular cytokine staining in B cells and monocytes. After 20 hours of pre‐incubation with 1 μg/ml CpG, PBMC were stimulated with 500 ng/ml ionomycin and 20 ng/ml phorbol 12‐myristate 13‐acetate for 4 hours in the presence of a Golgi inhibitor and subsequently stained intracellularly for TNF, IL‐6 and IL‐10. (A) Within the living cell population (as defined in supplementary figure 1), B cells were defined as CD19+. Their cytokine production was quantified using the mean fluorescence intensity (MFI) of the respective fluorescence labeled cytokine antibody (TNF ‐ A700, IL‐6 ‐ FITC, IL‐10 ‐ PE‐CF594). (B) Within the living cell population (as defined in supplementary figure 1), monocytes were defined as CD14+. Their cytokine production was quantified using the mean fluorescence intensity (MFI) of the respective fluorescence labeled cytokine antibody (TNF ‐ A700, IL‐6 ‐ FITC, IL‐10 ‐ PE‐CF594). Figure S3. Correlation between cellular composition of human peripheral blood mononuclear cells and patient related data. Immune cell frequencies in peripheral blood mononuclear cells of dimethyl fumarate treated (DMF; triangle) or control (circle) multiple sclerosis patients were correlated to (A) patient age, gender and expanded disability status scale (EDSS) score as well as (B) disease duration, premedication (interferon (IFN), glatiramer acetate (G [file BPA-29-640-s001.pdf]

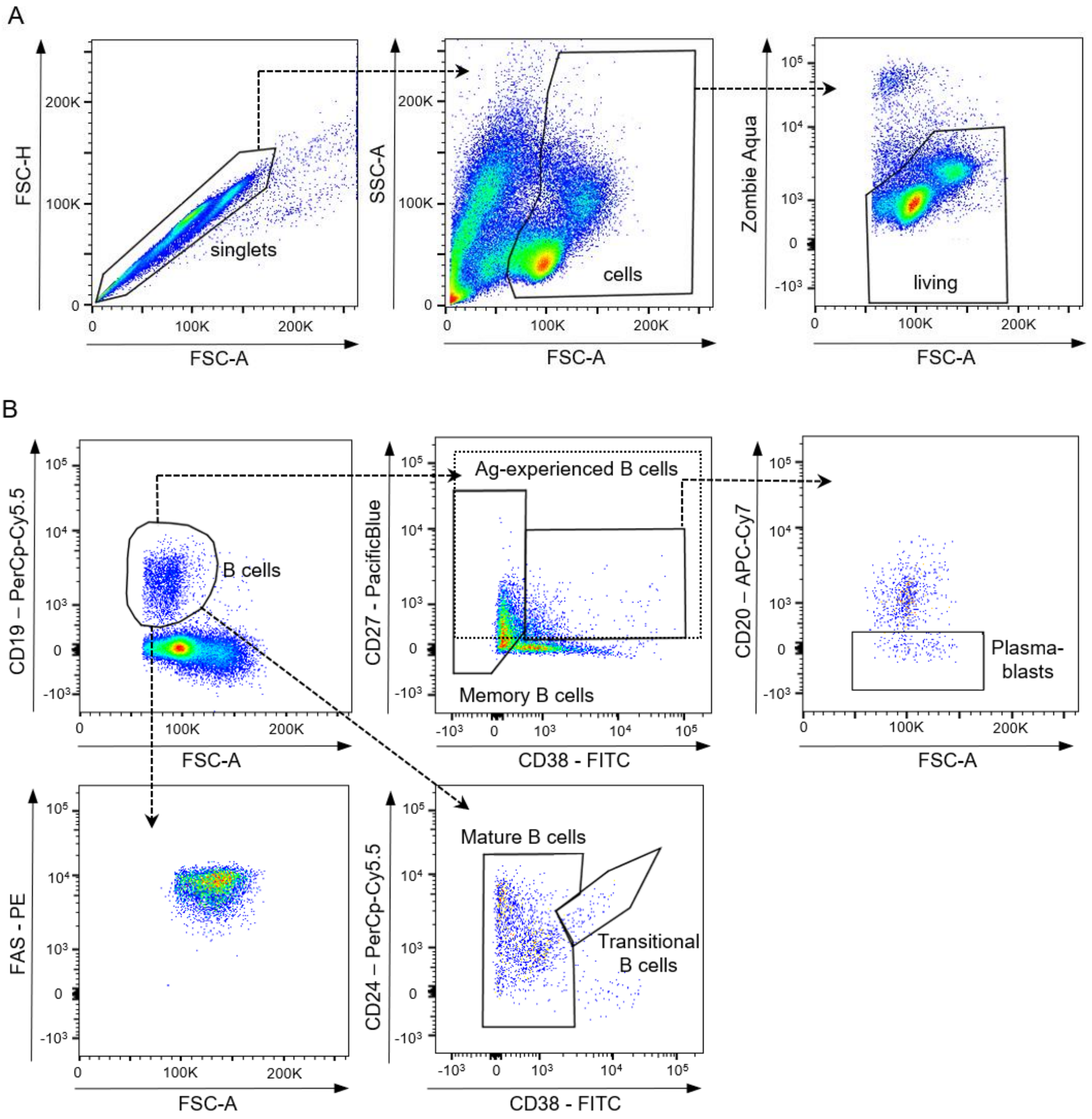

Supplementary figure 1: General pre-gating strategy and gating for B cell subsets and surface molecule expression. (**A**) Within all recorded events, doublets were excluded and living cells were determined using size exclusion and staining with Zombie-dye. (**B**) Within the living cell population (see A), B cells were defined as $CD19^+$. Surface marker were evaluated using the MFI. B cell subpopulations were identified as follows: Antigen- (Ag-) experienced B cells ($CD27^+$), memory B cells ($CD27^{var}$ $CD38^-$), plasmablasts ($CD20^-$ $CD27^+$ $CD38^+$), mature B cells ($CD24^{var}$ $CD38^{low}$) and transitional B cells ($CD24^{high}$ $CD38^{high}$).

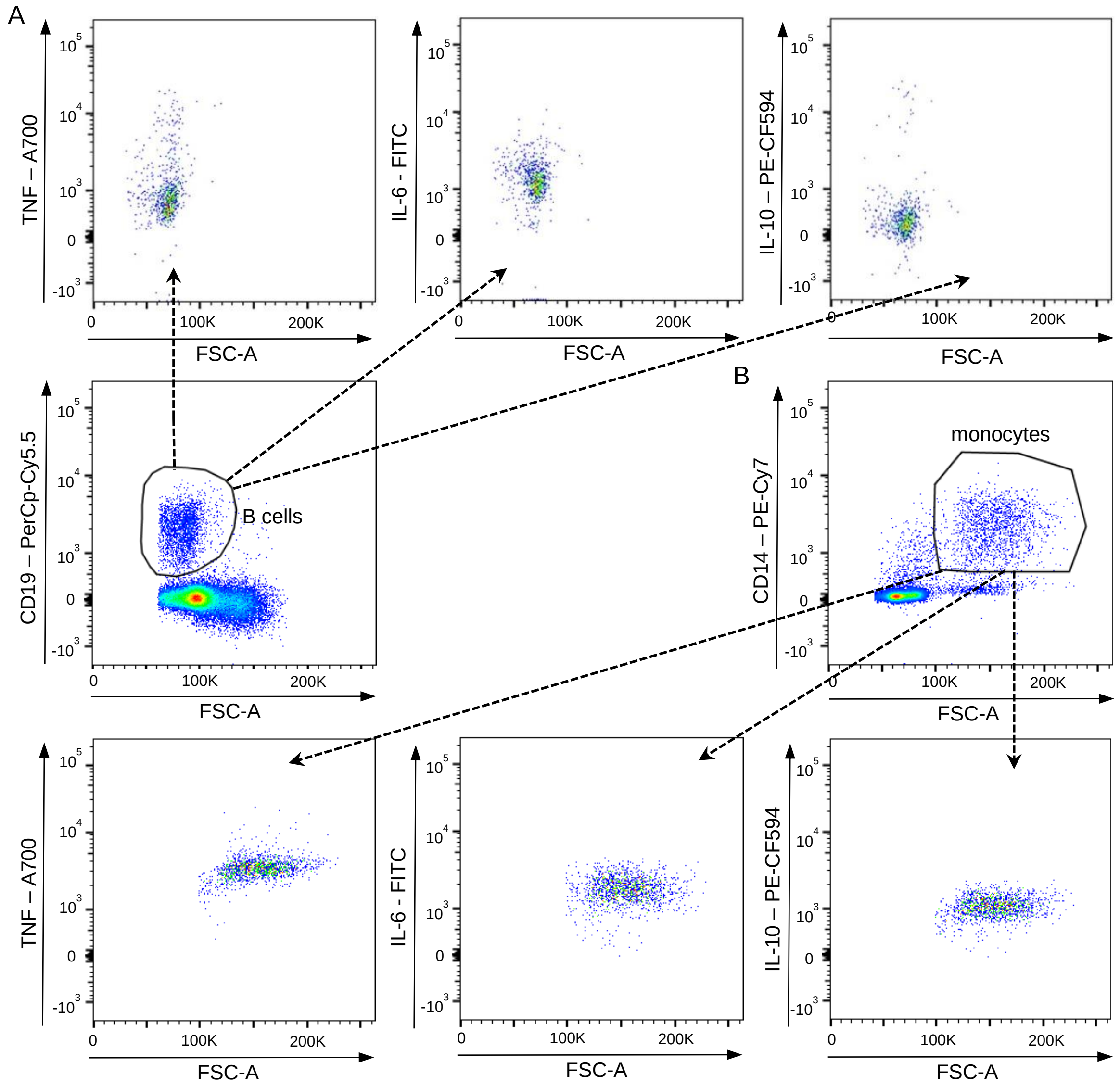

Supplementary figure 2: Gating strategy for intracellular cytokine staining in B cells and monocytes. After 20 hours of pre-incubation with 1 µg/ml CpG, PBMC were stimulated with 500 ng/ml ionomycin and 20 ng/ml phorbol 12-myristate 13-acetate for 4 hours in the presence of a Golgi inhibitor and subsequently stained intracellularly for TNF, IL-6 and IL-10. **(A)** Within the living cell population (as defined in supplementary figure 1), B cells were defined as $CD19^+$. Their cytokine production was quantified using the mean fluorescence intensity (MFI) of the respective fluorescence labeled cytokine antibody (TNF – A700, IL-6 – FITC, IL-10 – PE-CF594). **(B)** Within the living cell population (as defined in supplementary figure 1), monocytes were defined as $CD14^+$. Their cytokine production was quantified using the mean fluorescence intensity (MFI) of the respective fluorescence labeled cytokine antibody (TNF – A700, IL-6 – FITC, IL-10 – PE-CF594).

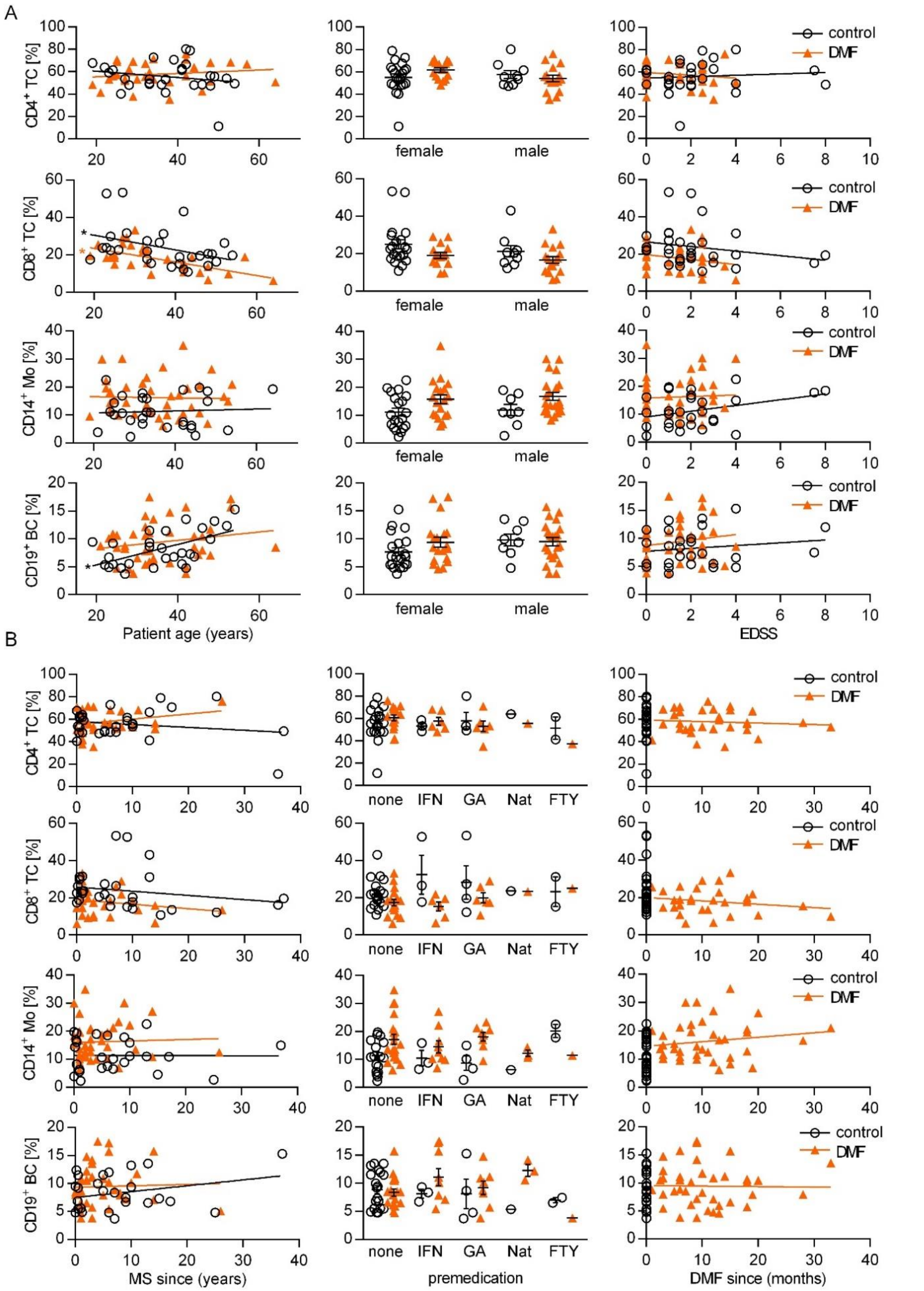

Supplementary figure 3: Correlation between cellular composition of human peripheral blood mononuclear cells and patient related data. Immune cell frequencies in peripheral blood mononuclear cells of dimethyl fumarate treated (DMF; triangle) or control (circle) multiple sclerosis patients were correlated to (A) patient age, gender and expanded disability status scale (EDSS) score as well as (B) disease duration, premedication (interferon (IFN), glatiramer acetate (GA), Natalizumab (Nat), fingolimod (FTY)) and treatment duration using linear regression (solid line; * = $p < 0.05$). Bars indicate mean ± standard error of the mean. BC = B cells, Mo = monocytes.

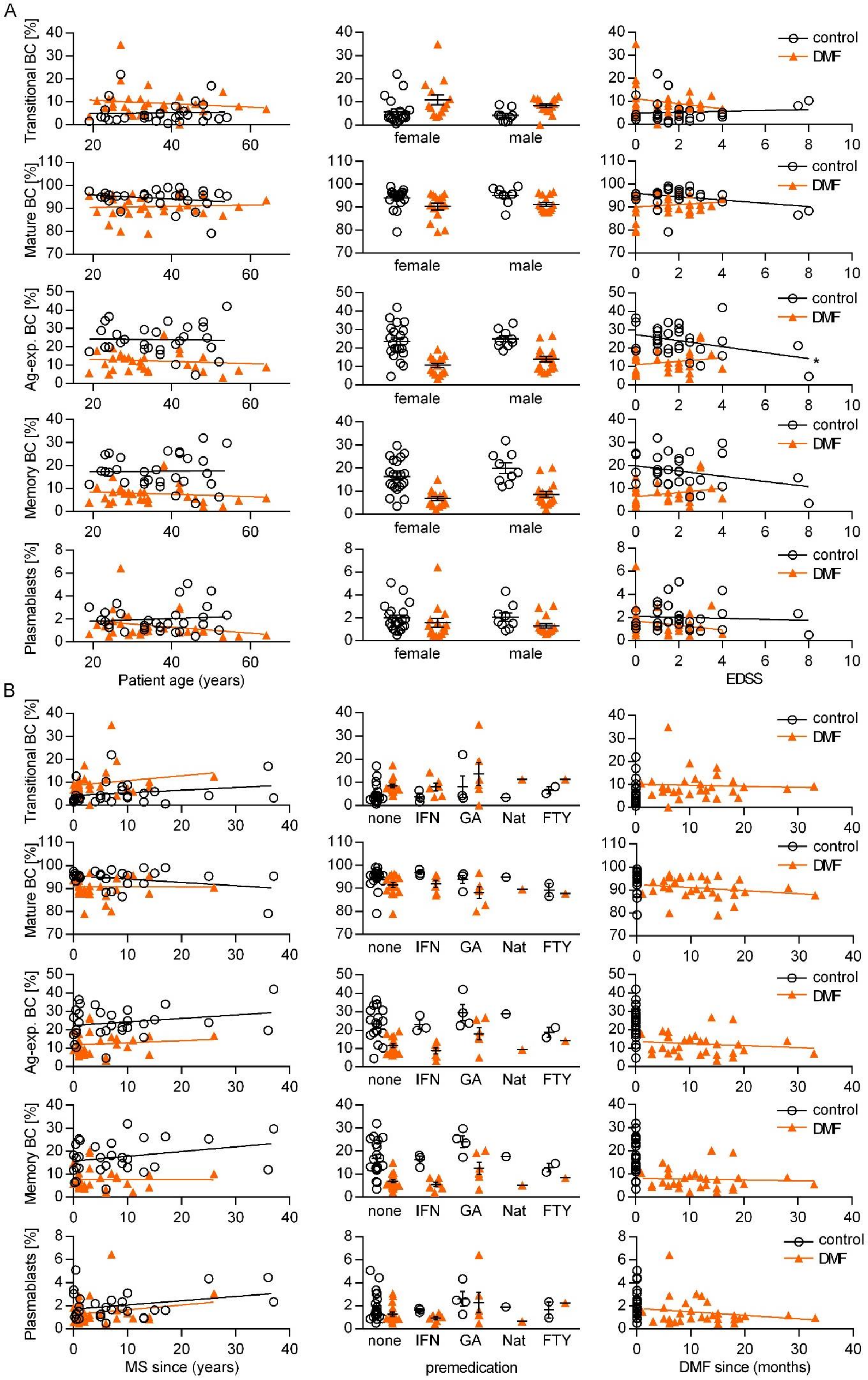
A
Transitional BC [%]
Mature BC [%]
Ag-exp. BC [%]
Memory BC [%]
Plasmablasts [%]
Patient age (years)
female
male
EDSS
control
DMF
*
B
MS since (years)
none
IFN
GA
Nat
FTY
premedication
DMF since (months)

Supplementary figure 4: Correlation between B cell subpopulations and patient related data. Transitional BC ($CD24^{high}$ $CD38^{high}$), mature BC ($CD24^{var}$ $CD38^{low}$), antigen-experienced BC ($CD27^{+}$; Ag-exp.), memory BC ($CD27^{var}$ $CD38^{-}$) and plasmablasts ($CD20^{-}$ $CD27^{+}$ $CD38^{+}$) were analyzed. B cell subpopulation frequencies of dimethyl fumarate treated (DMF; triangle) or control (circle) patients were correlated to (A) patient age, gender and expanded disability status scale (EDSS) score as well as (B) disease duration, premedication (interferon (IFN), glatiramer acetate (GA), Natalizumab (Nat), fingolimod (FTY)) and treatment duration using linear regression (solid line; * = $p < 0.05$). Bars indicate mean ± standard error of the mean. BC = B cells.

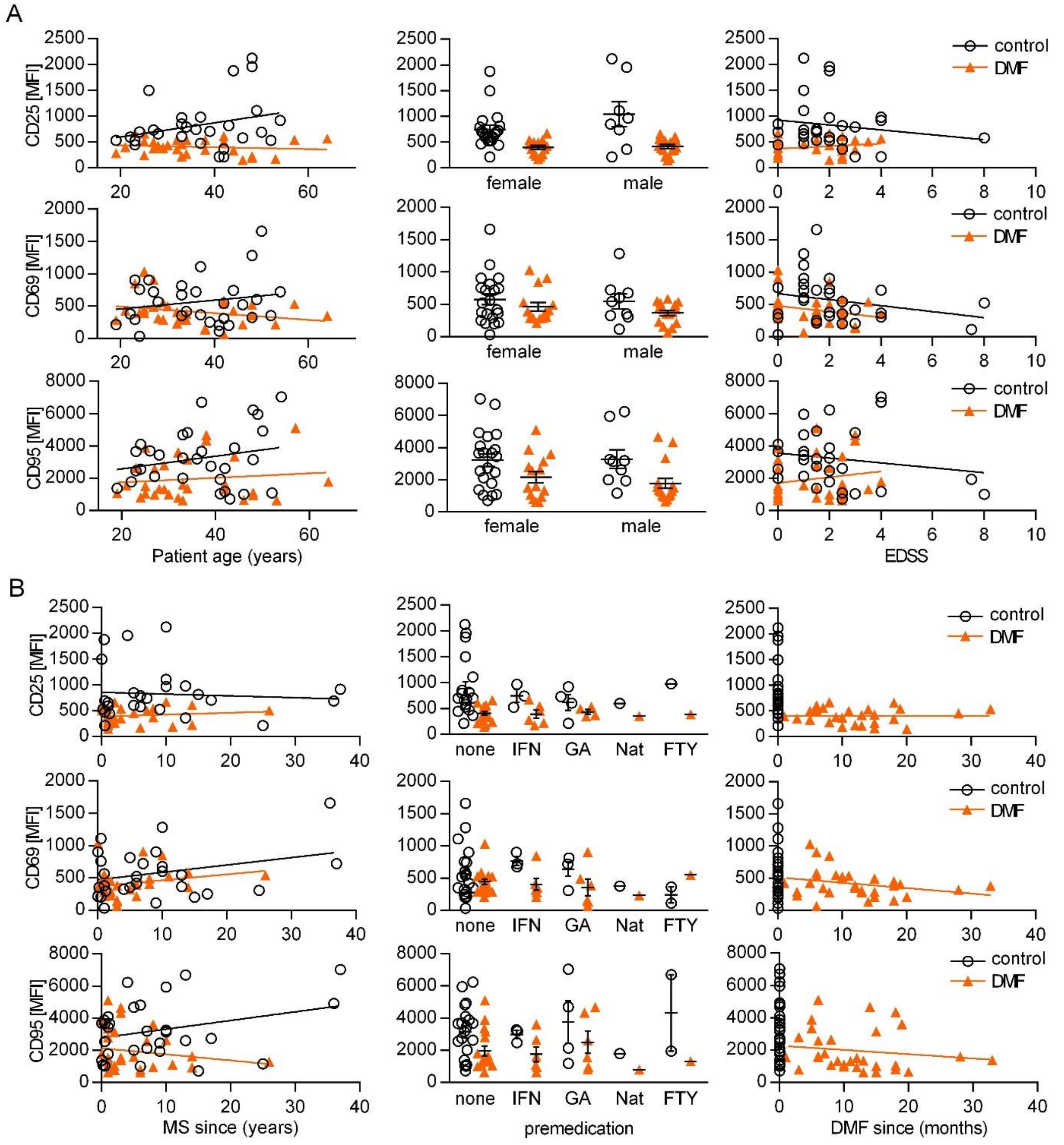

Supplementary figure 5: Correlation between B cell activation marker and patient-related data. Peripheral blood mononuclear cells were stimulated with 2µg/ml CpG for 20 hours. The expression of B cell activation marker (evaluated as mean fluorescent intensity: MFI) of dimethyl fumarate treated (DMF; triangle) or control (circle) patients were correlated to (A) patient age, gender and expanded disability status scale (EDSS) score as well as (B) disease duration, premedication (interferon (IFN), glatiramer acetate (GA), Natalizumab (Nat), fingolimod (FTY)) and treatment duration using linear regression (solid line; * = $p < 0.05$). Bars indicate mean ± standard error of the mean.

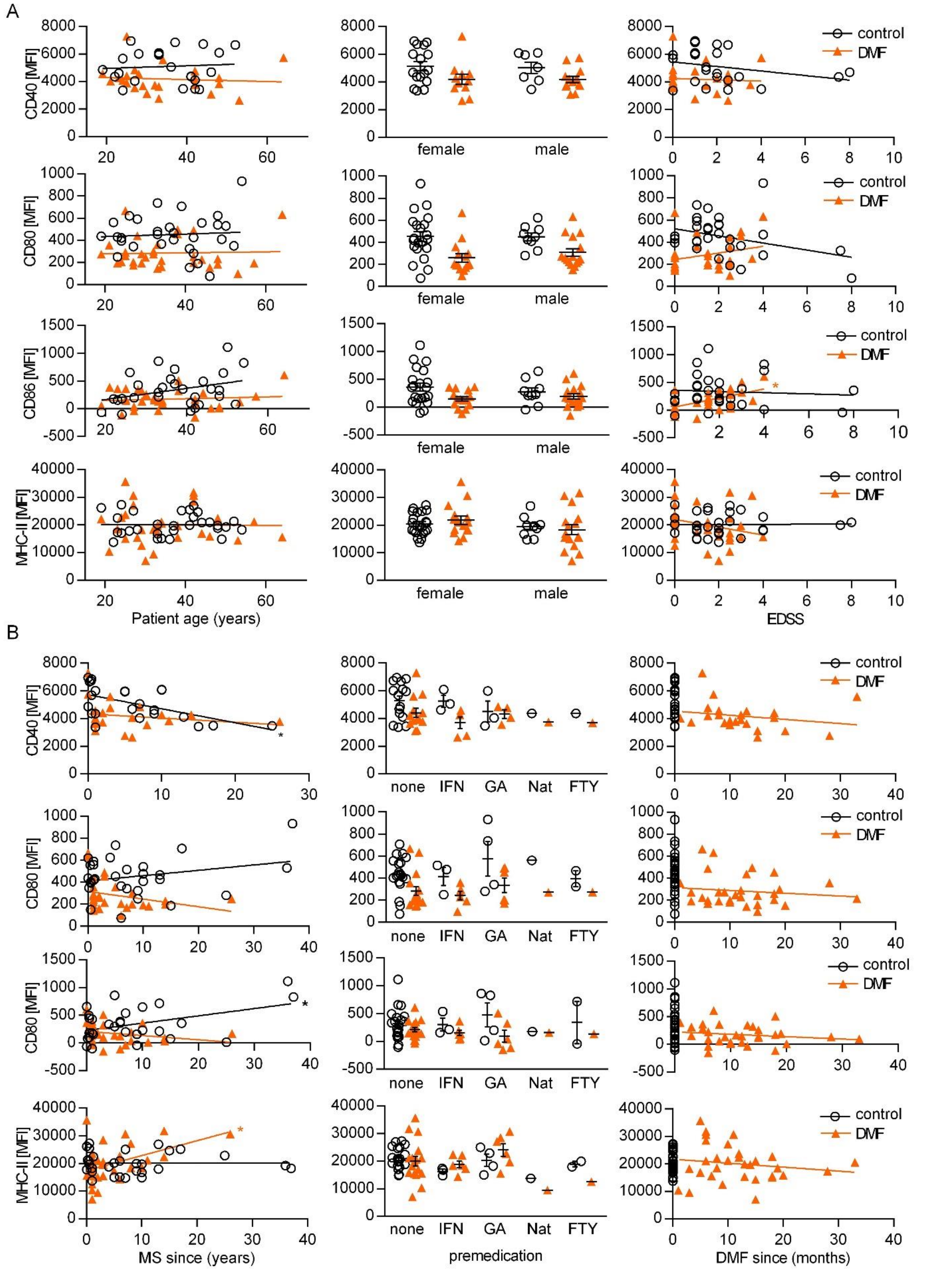

Supplementary figure 6: Correlation between antigen presentation-related B cell marker and patient related data. Peripheral blood mononuclear cells were stimulated with 2µg/ml CpG for 20 hours. The expression of antigen presentation-related B cell marker (evaluated as mean fluorescent intensity: MFI) of dimethyl fumarate treated (DMF; triangle) or control (circle) patients were correlated to (A) patient age, gender and expanded disability status scale (EDSS) score as well as (B) disease duration, premedication (interferon (IFN), glatiramer acetate (GA), Natalizumab (Nat), fingolimod (FTY)) and treatment duration using linear regression (solid line; * = $p < 0.05$). Bars indicate mean ± standard error of the mean.

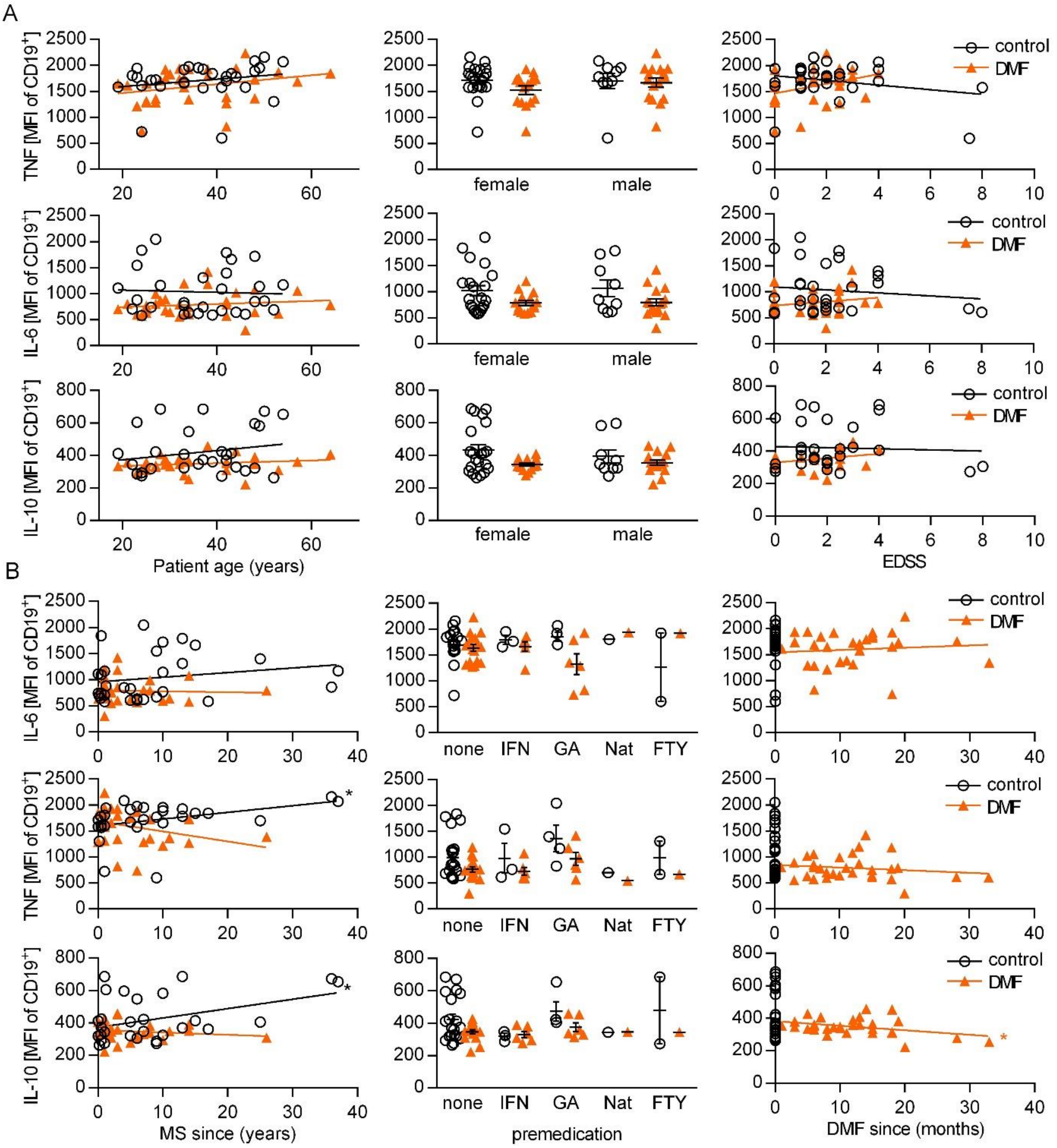

Supplementary figure 7: Correlation between B cell-produced cytokines and patient related data. After 20 hours of pre-incubation with 1 µg/ml CpG, peripheral blood mononuclear cells were stimulated with 500 ng/ml ionomycin and 20 ng/ml phorbol 12-myristate 13-acetate for 4 hours in the presence of a Golgi inhibitor and subsequently stained intracellularly for TNF, IL-6 and IL-10. Cytokines produced by $CD19^+$ B cells (evaluated as mean fluorescent intensity: MFI) of dimethyl fumarate treated (DMF; triangle) or control (circle) patients were correlated to (A) patient age, gender and expanded disability status scale (EDSS) score as well as (B) disease duration, premedication (interferon (IFN), glatiramer acetate (GA), Natalizumab (Nat), fingolimod (FTY)) and treatment duration using linear regression (solid line; * = $p < 0.05$). Bars indicate mean ± standard error of the mean.

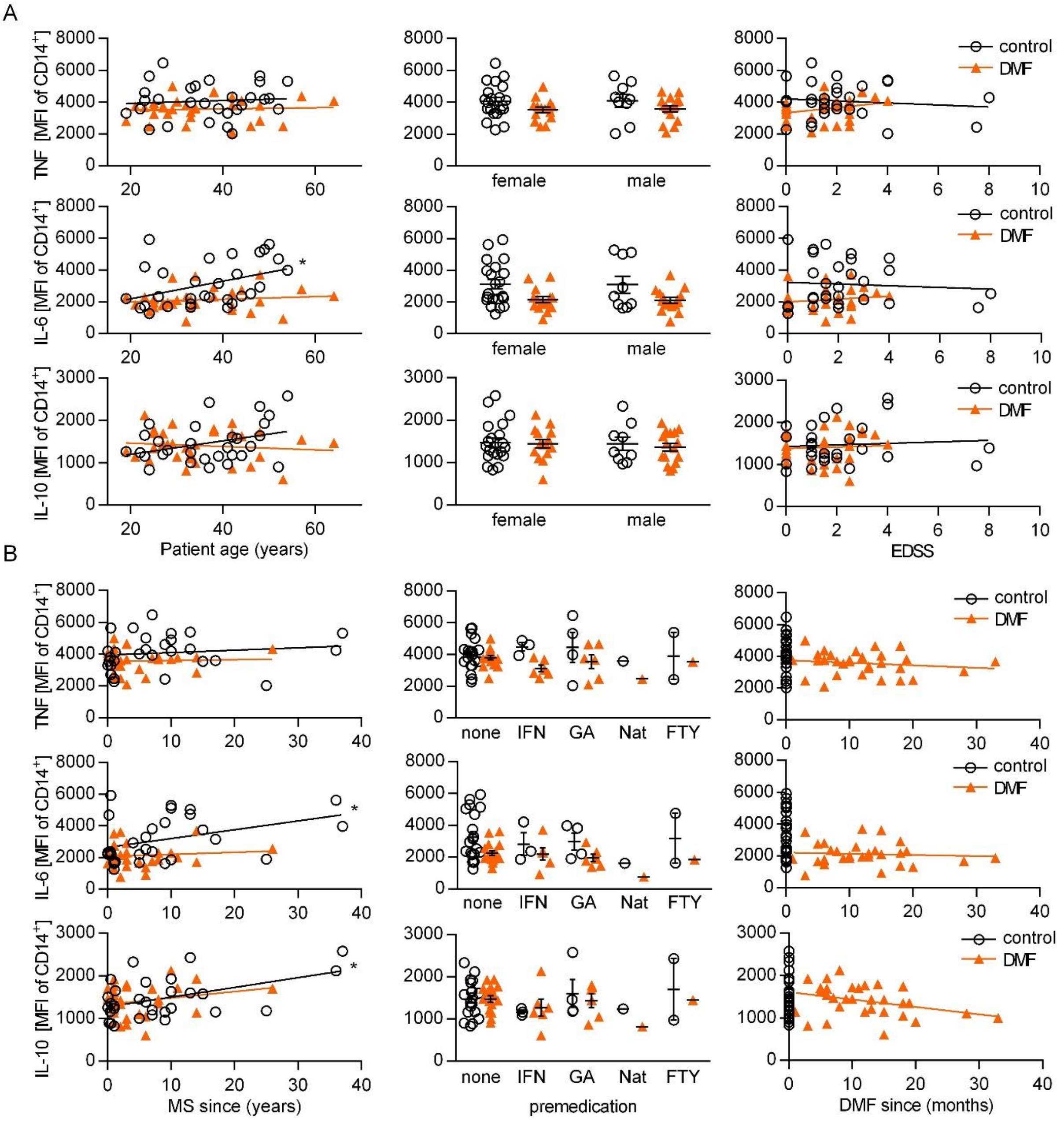

Supplementary figure 8: Correlation between monocyte-produced cytokines and patient related data. After 20 hours of pre-incubation with 1 µg/ml CpG, peripheral blood mononuclear cells were stimulated with 500 ng/ml ionomycin and 20 ng/ml phorbol 12-myristate 13-acetate for 4 hours in the presence of a Golgi inhibitor and subsequently stained intracellularly for TNF, IL-6 and IL-10. Cytokines produced by $CD14^+$ monocytes (evaluated as mean fluorescent intensity: MFI) of dimethyl fumarate treated (DMF; triangle) or control (circle) patients were correlated to (A) patient age, gender and expanded disability status scale (EDSS) score as well as (B) disease duration, premedication (interferon (IFN), glatiramer acetate (GA), Natalizumab (Nat), fingolimod (FTY)) and treatment duration using linear regression (solid line; * = $p < 0.05$). Bars indicate mean ± standard error of the mean.

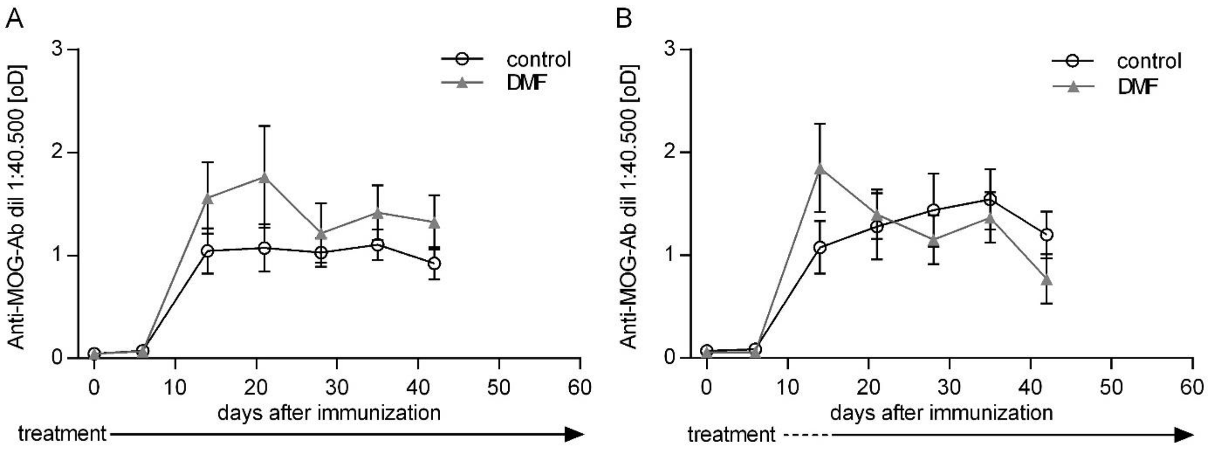

Supplementary figure 9: Preventive and therapeutic dimethyl fumarate treatment do not alter anti-MOG antibody levels in experimental autoimmune encephalomyelitis. (**A**) C57BL/6 mice were immunized with MOG protein$_{1\text{-}117}$ and treated with 15 mg/kg dimethyl fumarate (DMF) or vehicle (control) twice a day (d) from d -2 until d 60 post immunization (p.i.). Mean anti-MOG antibody levels in the serum ± standard error of the mean (SEM; n = 10 mice / group; *ns*; Mann-Whitney U test; data represent three independent experiments). (**B**) Therapeutic DMF treatment started when mice showed a score of 2 (hind limb weakness) or higher. Mean anti-MOG antibody levels in the serum ± standard error of the mean (SEM; n = 8-9 mice / group; *ns*; Mann-Whitney U test; data represent three independent experiments).

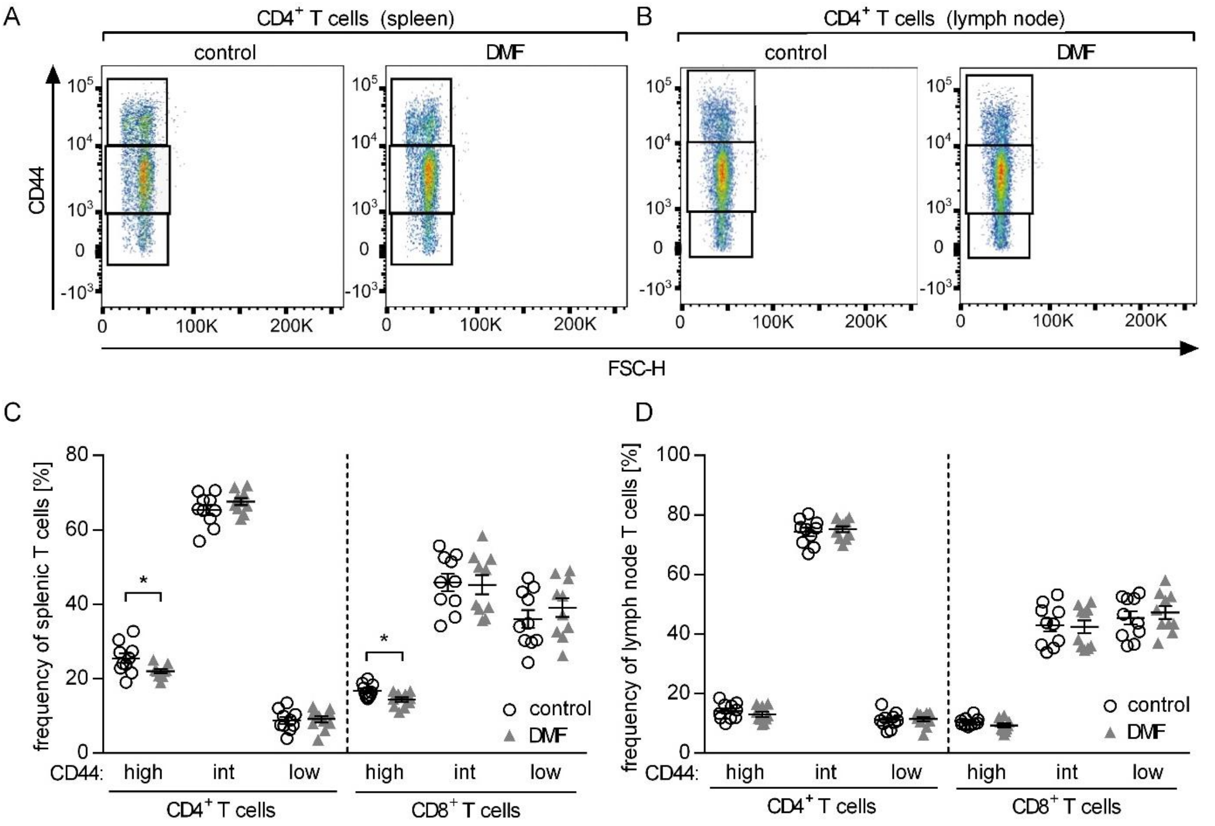

Supplementary figure 10: DMF treatment reduces T cell differentiation in the spleen. Mice were immunized with MOG protein$_{1\text{-}117}$ and treated with 15 mg/kg DMF or control twice a day from day (d)7 until d12 post immunization. (**A, B**) Representative dot plots of CD44 expression on $CD4^{+}$ T cells in spleen and lymph nodes. Frequency ± standard error of the mean of **(C)** splenic and **(D)** lymph node $CD4^{+}$ and $CD8^{+}$ T cells expressing high ($CD44^{hi}$), intermediate ($CD44^{int}$) and low ($CD44^{low}$) levels of CD44 (* = $p < 0.05$; unpaired t-test).

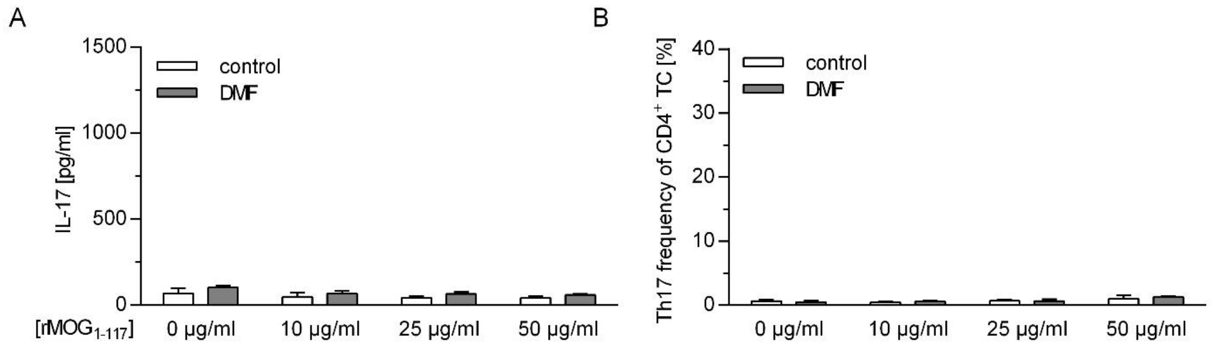

Supplementary figure 11: In vivo DMF-treated B cells do not alter Th17 differentiation. B cells were purified from mice immunized with MOG protein$_{1-117}$ and additionally treated with 15 mg/kg dimethyl fumarate (DMF) or control twice a day (d) from d2 until d12 post immunization. These isolated B cells were then co-cultured with CFSE-labeled 2D2 T cells and stimulated with 0, 25 or 50 µg/ml MOG protein$_{1-117}$ (rMOG$_{1-117}$). T cell proliferation was analyzed by flow cytometry and evaluated using the number of divisions. (**A, B**) IL-17 production of $CD4^+$ TC was analyzed by enzyme-linked immunosorbent assay and Th17 frequency was determined using intracellular flow cytometric staining at different concentrations of rMOG$_{1-117}$. (n = 4 mice / group; * = $p < 0.05$, ** = $p < 0.01$, *** = $p < 0.001$; unpaired t-test).

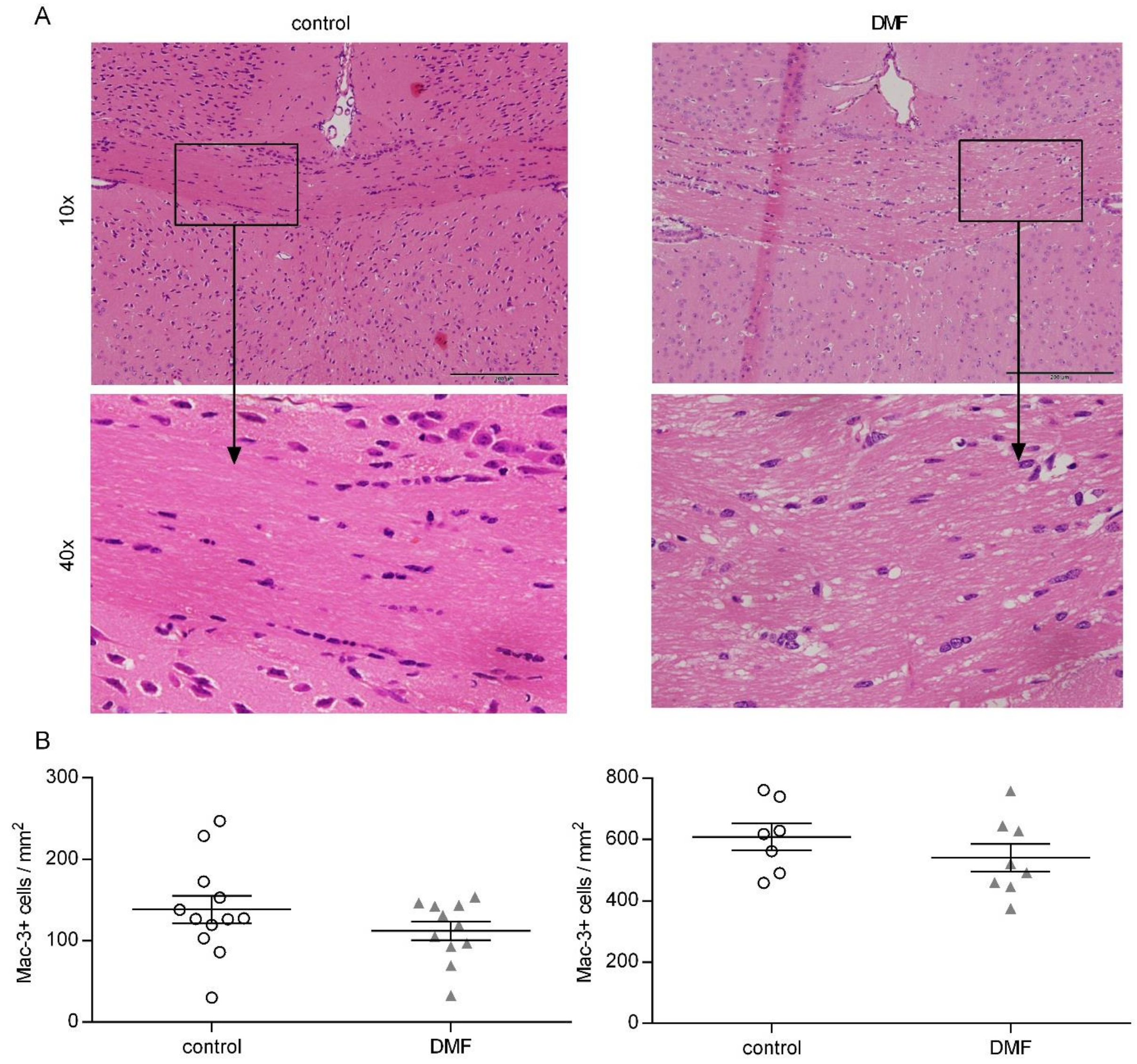

Supplementary figure 12: DMF treatment did not alter macrophage infiltration after long-term cuprizone diet. C57BL/6 mice were fed with 0.25% cuprizone and treated with 15 mg/kg DMF or vehicle (control) twice a day for six weeks. (**A**) Hematoxylin and eosin stain of the corpus callosum is presented. (**B**) Mac-3^{+} cells in the corpus callosum after seven days (left graph) and six weeks (right graph) of DMF/control treatment in the cuprizone model (mean ± SEM; ns; unpaired t-test).

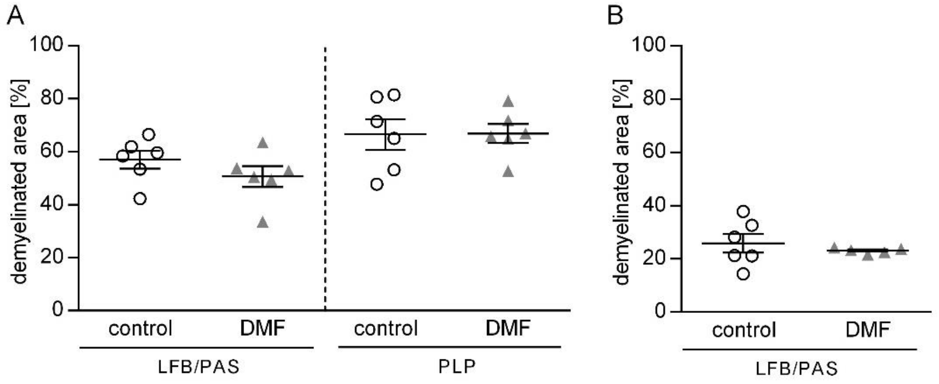

Supplementary figure 13: Demyelination of the corpus callosum was not majorly altered in preventative DMF treatment after long-term cuprizone diet. (**A**) C57BL/6 mice were fed with 0.25% cuprizone and treated with 15 mg/kg DMF or vehicle (control) twice a day for six weeks in a preventive setting. LFB/PAS and PLP staining was performed to determine the percentage of demyelinated area of the corpus callosum shown as mean ± SEM (ns; unpaired t-test). (**B**) In an interventional setting, C57BL/6 mice were fed with 0.25% cuprizone for five weeks. Afterwards, cuprizone diet was stopped and mice were treated with 15 mg/kg DMF or control twice a day for three days. LFB/PAS staining was performed to determine the percentage of demyelinated area of the corpus callosum shown as mean ± SEM (ns; unpaired t-test).

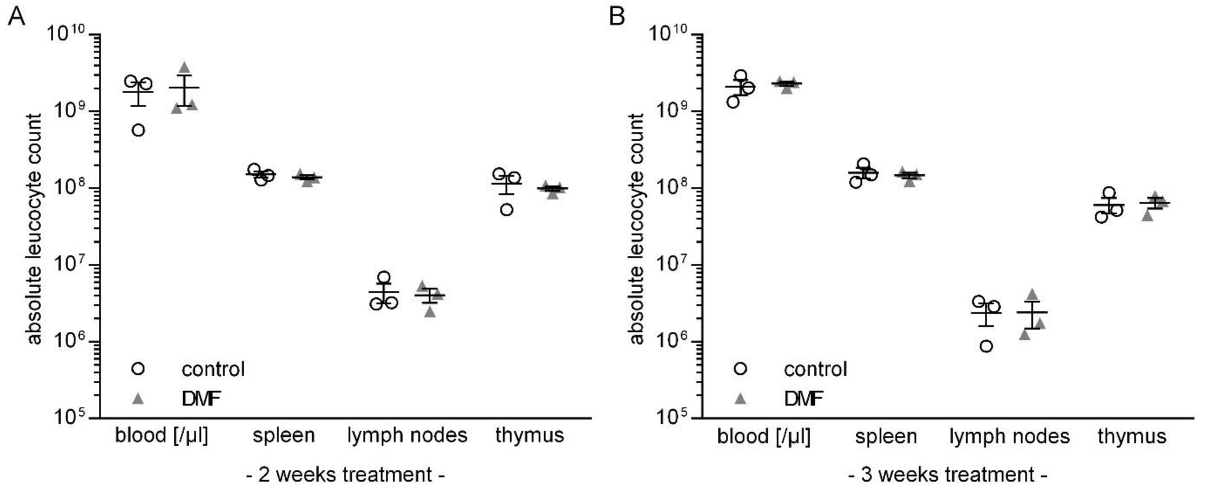

Supplementary figure 14: DMF treatment did not change absolute leukocyte counts in different organs. Mice were immunized with MOG protein$_{1-117}$ and treated with 15 mg/kg DMF or control twice a day from day (d)7 until d12 post immunization (**A**) Absolute leukocyte counts of the blood, the spleen, the lymph nodes and the thymus were determined after two weeks of DMF/control treatment. (**B**) Absolute leukocyte counts of the blood, the spleen, the lymph nodes and the thymus were determined after three weeks of DMF/control treatment (mean ± SEM; ns; unpaired t-test).

Supplementary table 1: Immune cell frequencies in blood (A), spleen (B) and lymph nodes (C) of mice are altered upon dimethyl fumarate treatment. C57BL/6 mice were immunized with MOG $protein_{1-117}$ and treated with 15 mg/kg dimethyl fumarate DMF or vehicle (control) twice a day (d) from d -7 until d12 post immunization. Within all leukocytes, mean cell frequencies of $CD4^+$ T cells, $CD8^+$ T cells, $CD19^+$ B cells and $CD11b^+$ myeloid cells are shown. Data represent two independent experiments (* = $p < 0.05$; unpaired t-test).

**A) blood**	control	DMF	p
$CD4^+$ T cells	14.7% (±1.8%)	12.6% (±1.5%)	0.0289
$CD8^+$ T cells	8.4% (±1.5%)	7.9% (±1.8%)	0.5733
$CD19^+$ cells	31.3% (±6.3%)	28.5% (±7.1%)	0.4336
$CD11b^+$ cells	40.8% (±8.3%)	34.8% (±16.5%)	0.3917
**B) spleen**			
$CD4^+$ T cells	12.8% (±2.4%)	15.4% (±1.9%)	0.0157
$CD8^+$ T cells	7.1% (±1.8%)	8.9% (±1.3%)	0.0178
$CD19^+$ cells	42.9% (±4.7%)	42.6% (±3.3%)	0.9002
$CD11b^+$ cells	20.4% (±3.0%)	21.2% (±5.3%)	0.7738
**C) lymph nodes**			
$CD4^+$ T cells	20.0% (±2.7%)	23.2% (±1.0%)	0.0242
$CD8^+$ T cells	13.2% (±2.7%)	14.9% (±2.3%)	0.1486
$CD19^+$ cells	60.3% (±1.9%)	57.6% (±3.7%)	0.0729
$CD11b^+$ cells	1.6% (±0.9%)	1.5% (±0.7%)	0.9348
